# Supplementary material for: Environment-sensitive emission of anionic hydrogen-bonded urea-derivative–acetate-ion complexes and their aggregation-induced emission enhancement
Source: Commun Chem. 2021 Dec 2;4:168. doi: 10.1038/s42004-021-00601-3 (PMC9814938; doi:10.1038/s42004-021-00601-3)
Supplement: Supplementary file 1 — Description of Additional Supplementary Files [file 42004_2021_601_MOESM1_ESM.pdf]

Description of Additional Supplementary Files

File Name: Supplementary Data 1

Description: Crystallographic information file for p-2Urea.

File Name: Supplementary Data 2

Description: Optimized Cartesian coordinates and total energies of molecules and complexes.
