# Supplementary material for: Environment-sensitive emission of anionic hydrogen-bonded urea-derivative–acetate-ion complexes and their aggregation-induced emission enhancement
Source: Commun Chem. 2021 Dec 2;4:168. doi: 10.1038/s42004-021-00601-3 (PMC9814938; doi:10.1038/s42004-021-00601-3)
Supplement: Supplementary file 2 — Supplementary Information [file 42004_2021_601_MOESM2_ESM.pdf]

Environment-sensitive emission of anionic hydrogen-bonded urea-derivative–acetate-ion complexes and their aggregation-induced emission enhancement

Supplementary Information

Masaki Takahashi*, Nozomu Ito, Naoki Haruta, Hayato Ninagawa, Kohei Yazaki, Yoshihisa Sei, Tohru Sato, and Makoto Obata

Table of Contents:

1. Supplementary Methods.. S2
2. Supplementary Tables and Figures S4
3. Supplementary References.. S32

1. Supplementary Methods

Supplementary Method 1. Synthesis of 1,8-bis(4'-anilino)naphthalene[1]. A solution of 1,8-diiodonaphthalene (490 mg, 1.29 mmol), 4-aminophenylboronic acid pinacol ester (726 mg, 3.31 mmol), trans-$(Cy_2NH)_2Pd(OAc)_2$ (195 mg, 0.332 mmol), and NaOH (239 mg, 5.98 mmol) was stirred in 9 mL of an ethanol:toluene:water (1:1:1) solution at 95 °C under N_2 for 24 h. The resulting mixture was allowed to reach room temperature before undergoing celite filtration. The resulting reaction mixture was extracted with ethyl acetate three times and washed with water and brine. The separated organic layer was dried with anhydrous Na_2SO_4 and the solvent was removed under reduced pressure. The residue was purified via silica-gel column chromatography using an ethyl acetate:ethanol = 95:5 solution as the eluent to yield the product as a brown viscous liquid (266 mg, 0.857 mmol, 67% yield). This material was used in the following step without further purification. Synthesis of ***p*-2Urea**. A solution of 1,8-bis(4'-anilino)naphthalene (195 mg, 0.628 mmol) and 4-tert-butylphenyl isocyanate (339 mg, 1.93 mmol) was stirred into 20 mL of tetrahydrofuran:N,N-dimethylformamide (3:1) at room temperature under N_2 for 4 h. The resulting mixture was extracted with ethyl acetate three times and washed with water and brine. The separated organic layer was dried with anhydrous Na_2SO_4, and the solvent was removed under reduced pressure. The residue was collected by filtration and washed with acetone to yield ***p*-2Urea** as a white powder (316 mg, 0.478 mmol, 75% yield). ^{1}H NMR (500 MHz, DMSO-d_6): δ 8.40 (d, 10 Hz, 4H), 8.00 (d, 9.5 Hz, 2H), 7.59 (t, 15.5 Hz, 2H), 7.38 (d, 8Hz, 2H), 7.29 (d, 9.0 Hz, 4H), 7.23 (d, 7 Hz 4H), 7.07 (d, 8.5 Hz,4H), 6.85 (d, 8.5 Hz, 4H), 1.24 (s,18H) ppm; ^{13}C NMR (500 MHz, DMSO-d_6): δ 152.54, 144.10, 139.96, 137.72, 137.22, 136.32, 135.43, 130.72, 129.81, 128.79, 128.24, 125.43, 118.23, 116.83, 34.00, 31.42 ppm. Single crystals of *p-2Urea* for single-crystal X-ray diffraction analysis were obtained by vapour diffusion of diethyl ether into a DMSO solution.

Supplementary Method 2. Synthesis of 1,8-bis(3'-anilino)naphthalene. A solution of 1,8-diiodonaphthalene (490 mg, 1.29 mmol), 3-aminophenylboronic acid pinacol ester (726 mg, 3.31 mmol), trans-$(Cy_2NH)_2Pd(OAc)_2$ (194 mg, 0.330 mmol), and NaOH (250 mg, 6.25 mmol) were stirred into 9 mL of an ethanol:toluene:water (1:1:1) solution at 95 °C under N_2 for 24 h. The resulting mixture was allowed to reach room temperature and then underwent celite filtration. The resulting reaction mixture was extracted with ethyl acetate three times and washed with water and brine. The separated organic layer was dried with anhydrous Na_2SO_4

and the solvent was removed under reduced pressure. The residue was purified via silica-gel column chromatography using an ethyl acetate:ethanol = 95:5 solution as the eluent to yield the product as a brown viscous liquid (270 mg, 0.869 mmol, 67% yield). This material was used in the following step without further purification. Synthesis of ***m*-2Urea**. A solution of 1,8-bis(3'-anilino)naphthalene (234 mg, 0.754 mmol) and 4-tert-butylphenyl isocyanate (253 mg, 1.45 mmol) was stirred into 7.2 mL of dehydrated tetrahydrofuran at 76 °C under N_2 overnight. After the solvent was removed under reduced pressure, the residue was collected by filtration and washed with acetone to yield ***m*-2Urea** as a white powder (290 mg, 0.438 mmol, 58% yield). ^{1}H NMR (500 MHz, DMSO-d_6): δ8.46 (s, 1H), 8.31 (s, 1H), 8.22 (s, 2H), 8.06 (d, J = 8.2 Hz, 2H), 7.62 (t, J = 7.8 Hz, 2H), 7.43 (d, J = 6.9 Hz, 2H), 7.34 (d, J = 8.5 Hz, 2H), 7.27 (d, J = 8.5 Hz, 2H), 7.13-7.19 (m, 5H), 7.00-6.93 (m, 4H), 6.90 (s, 1H), 6.83 (t, J = 7.7 Hz, 1H), 6.76 (d, J = 6.7 Hz, 1H), 6.56 (d, J = 7.5 Hz, 1H), 1.25 (s, 9H), 1.23 (s, 9H) ppm; ^{13}C NMR (500 MHz, DMSO-d_6): δ152.86, 152.81, 144.45, 144.24, 143.32, 140.44, 138.90, 138.81, 137.61, 137.43, 135.59, 130.95, 130.84, 128.96, 127.98, 127.72, 125.83, 125.80, 125.59, 122.98, 122.87, 120.20, 119.70, 118.58, 118.38, 116.07, 34.34, 34.27, 31.72 ppm; HRMS (ESI) exact mass calculated for [M]+ ($C_{44}H_{44}N_4O_2$) requires *m*/*z* 660.3464, found *m*/*z* 660.3459.

***p*-1Urea** was synthesised by following the method reported in our previous paper[2].

Supplementary Method 3. Computational details. Gaussian16 was used for all geometry optimisations[3]. The geometry of *p*-2Urea, *p*-1Urea, and *m*-2Urea were first optimised at the B3LYP/6-31G (d, p) before TF-DFT excitation energy calculation were carried out at the CAM-B3LYP/6-31G+ (d) level of theory[4, 5]. The geometry of the complex of *p*-2Urea, *p*-1Urea, and *m*-2Urea with two equivalents of AcO– were first optimised at the CAM-B3LYP/6-31G+ (d) level of theory before TD-DFT excitation energy calculations were carried out at the CAM-B3LYP/6-31G+ (d) level. In addition, for discussing the theoretical origin of AIEE, *p*-2Urea dimers were optimised at the B3LYP/3-21G level of theory with the Grimme's empirical dispersion D3, and then single-point excited state calculations were performed at the S_0-optimised structure at the CAM-B3LYP/3-21G level of theory with the Grimme's empirical dispersion D3. All the minimum-energy structures were verified by frequency analyses.

2. Supplementary Tables and Figures

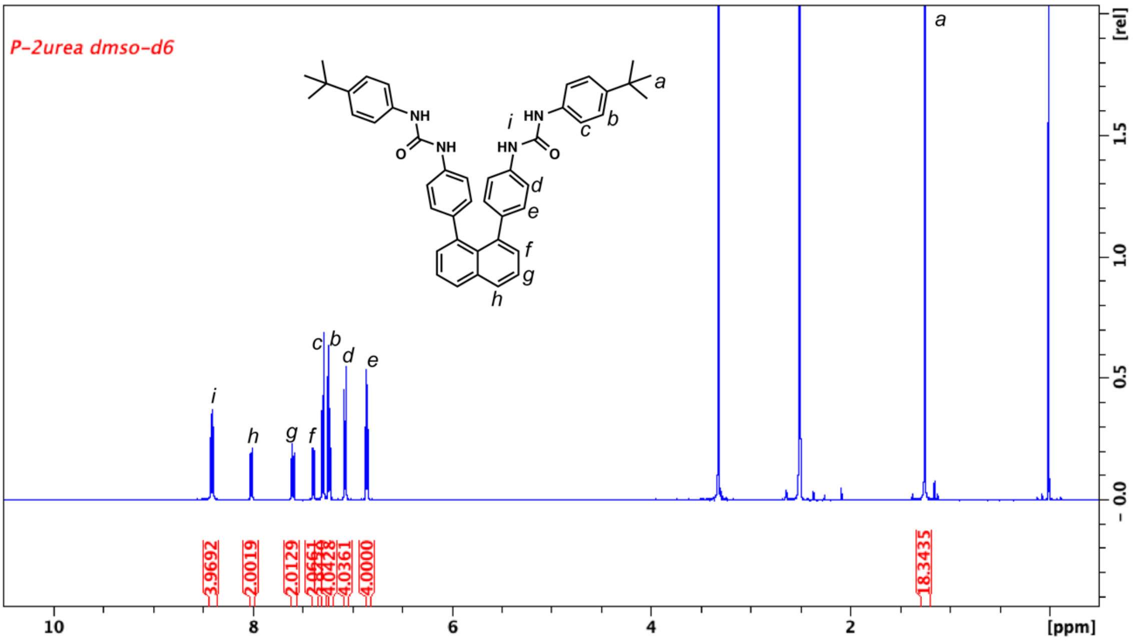

Supplementary Figure 1. ^{1}H NMR spectrum of ***p*-2Urea** in DMSO-d_6.

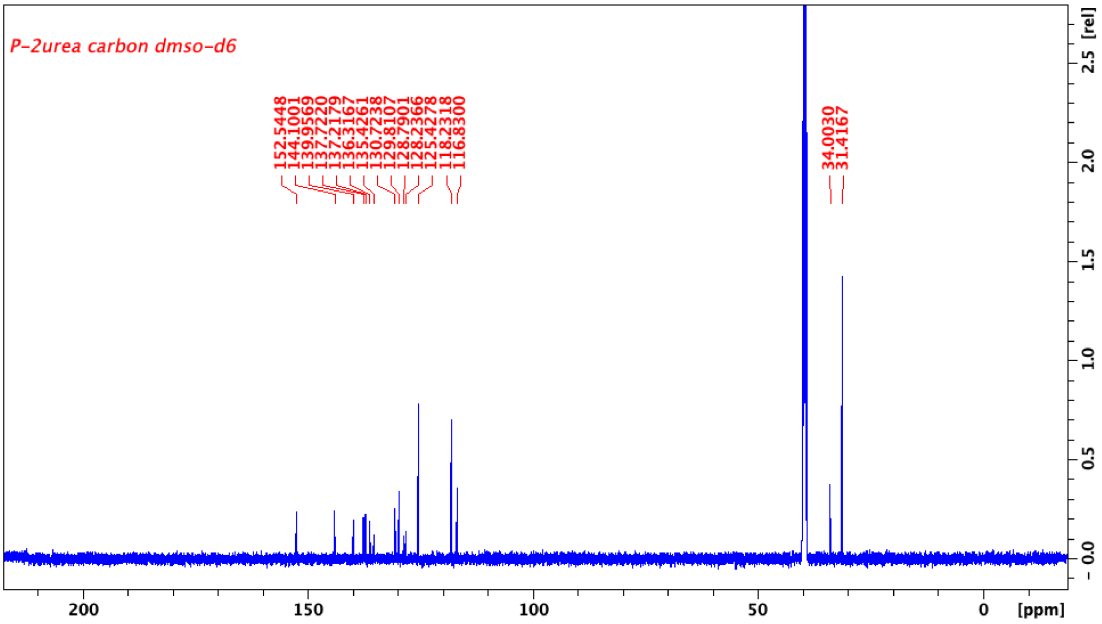

Supplementary Figure 2. ^{13}C NMR spectrum of ***p*-2Urea** in DMSO-d_6.

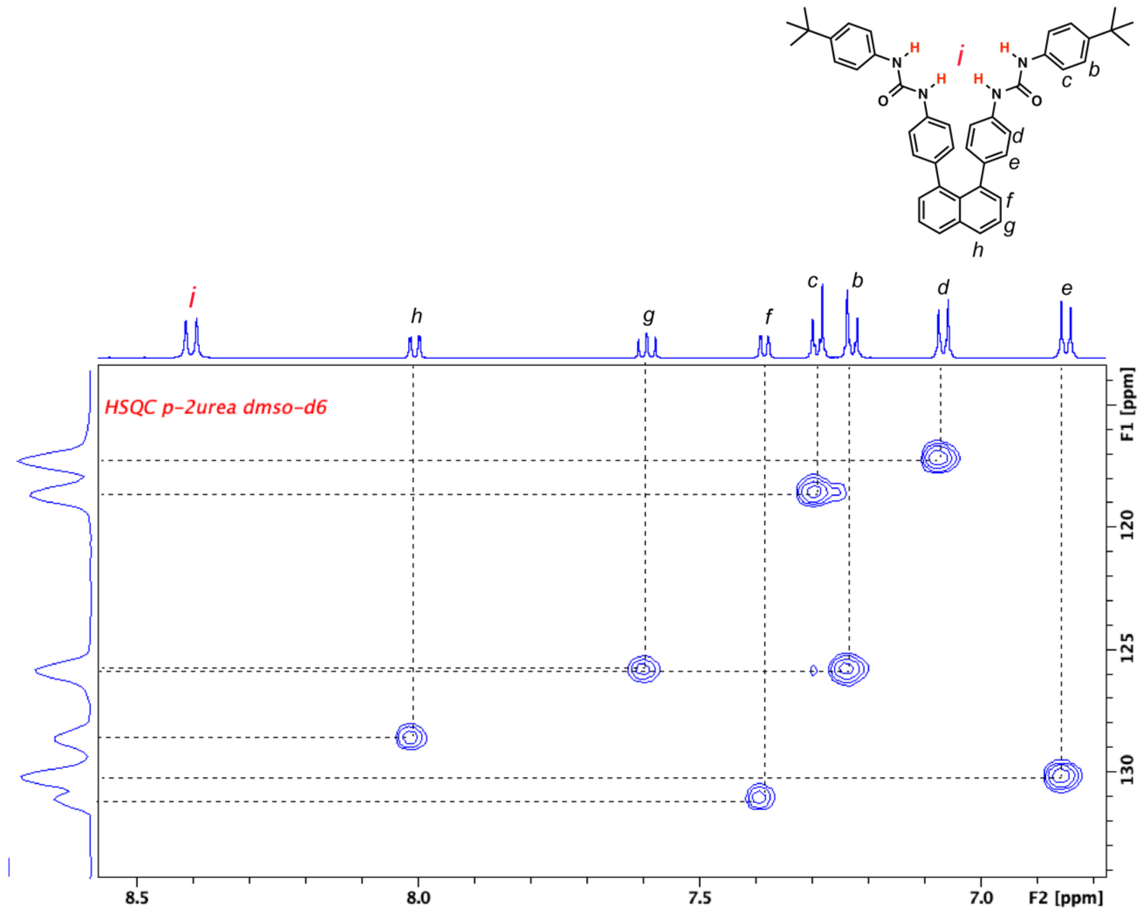

Supplementary Figure 3. Heteronuclear single quantum correlation (HSQC) spectrum of ***p*-2Urea** in DMSO-d_6.

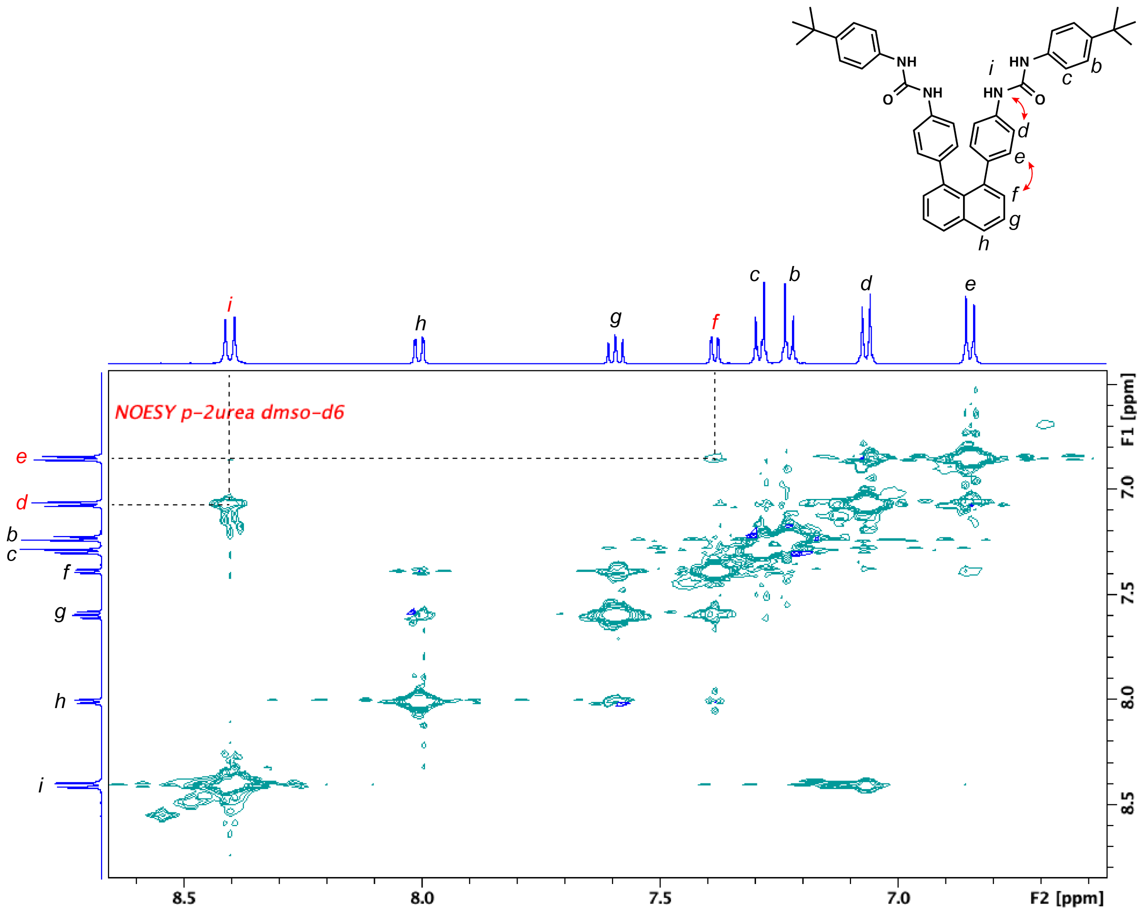

Supplementary Figure 4. Nuclear Overhauser effect spectroscopy (NOESY) results for ***p*-2Urea** in DMSO-d_6.

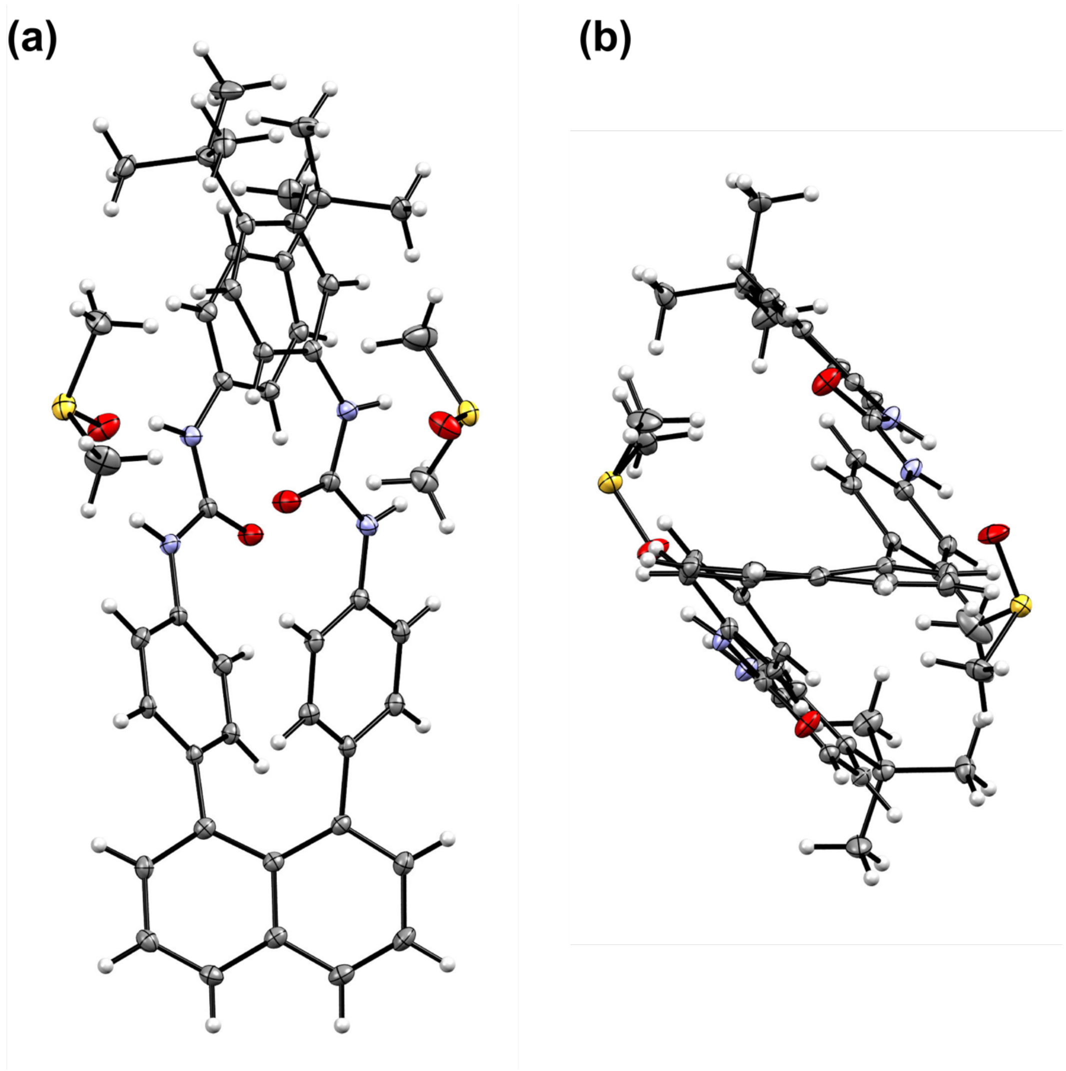

Supplementary Figure 5. Molecular structure of ***p*-2Urea**. (a) Top and (b) side views of the molecular structure of ***p*-2Urea** hydrogen-bonded to the S=O groups of DMSO. Thermal ellipsoids are drawn at the 50% probability level.

Supplementary Table 1. Crystal data and structure refinement for ***p*-2Urea**-DMSO solvate.

Chemical formula Sum	$C_{48}H_{56}N_4O_4S_2$
Formula weight	817.08 g/mol
Temperature	90 K
Crystal system	Orthorhombic
Space group	$Pna2_1$
a	21.163(3) Å
b	15.142(4) Å
c	13.580(2) Å
α	90°
β	90°
γ	90°
Volume	4351.9(12) $Å^3$
Z	4
Reflection	25690
Unique reflection	9232
Goodness-of-fit on F^2	0.925
Final R indices [$I > 2\sigma(I)$], R1, wR2	R1 = 0.0338, wR2 = 0.0894
R indices (all data), R1, wR2	R1 = 0.0368, wR2 = 0.0920

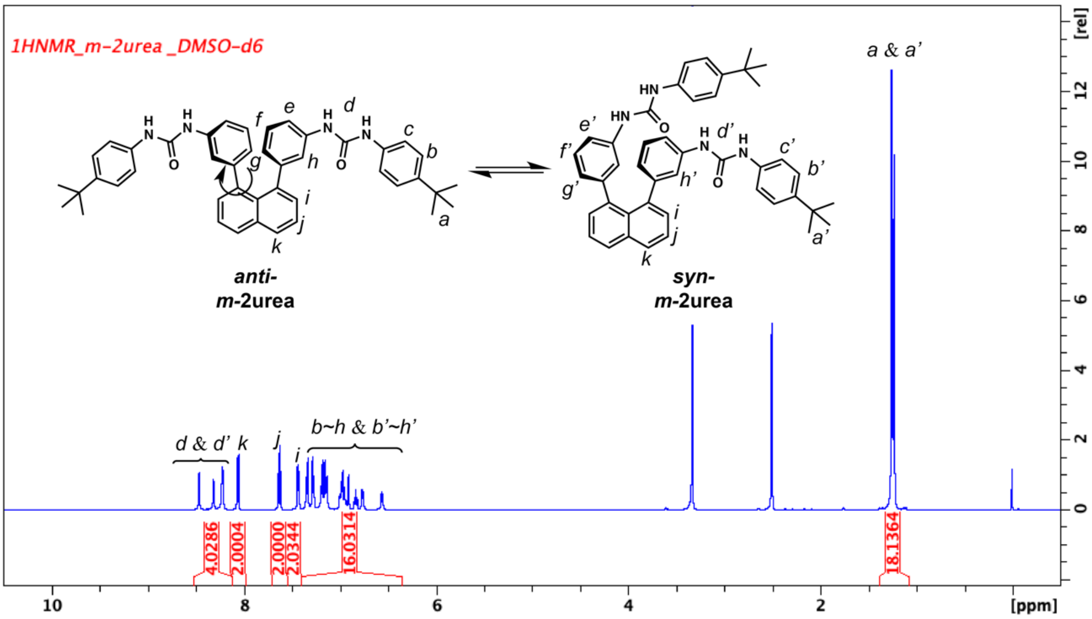

Supplementary Figure 6. ^{1}H NMR spectrum of ***m*-2Urea** in DMSO-d_6.

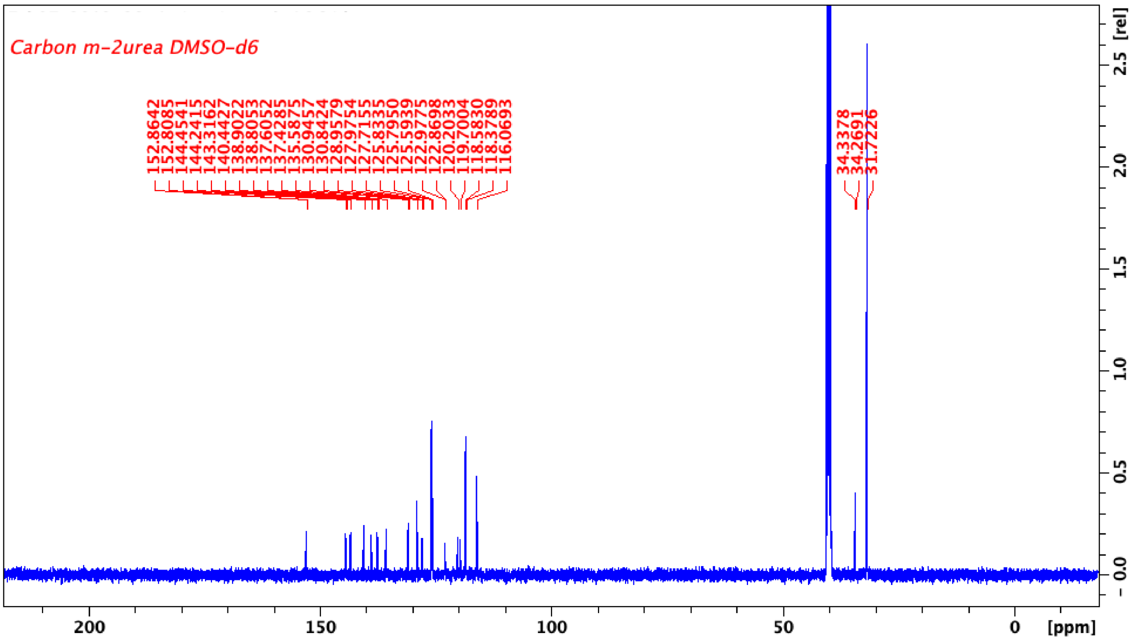

Supplementary Figure 7. ^{13}C NMR spectrum of ***m*-2Urea** in DMSO-d_6.

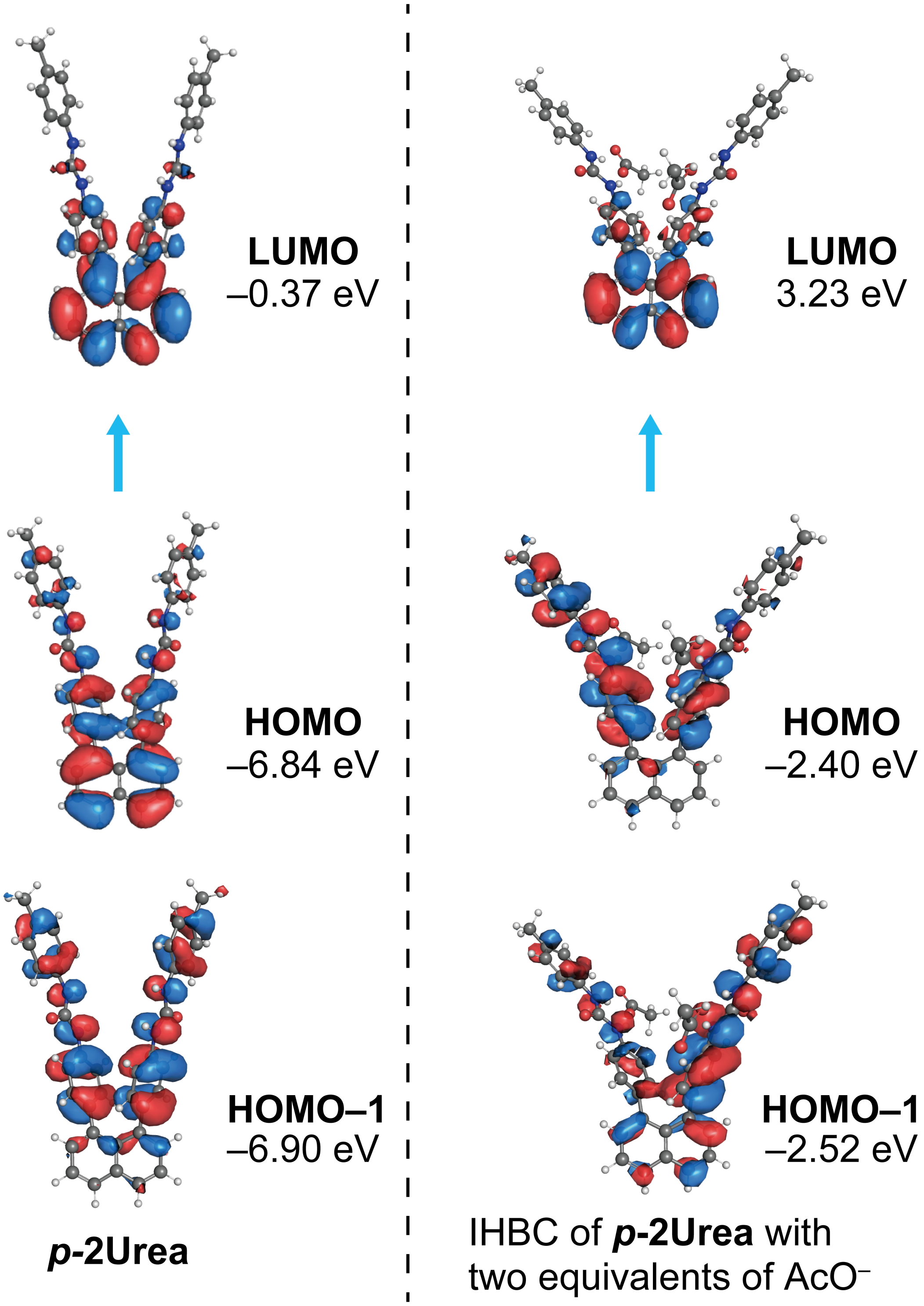

Supplementary Figure 8. Frontier molecular orbital amplitude plots of the HOMOs and LUMOs of ***p*-2Urea** (left) and the complex of ***p*-2Urea** with two equivalents of AcO^- (right). The geometry of ***p*-2Urea** was first optimised at the B3LYP/6-31G (d, p) before TF-DFT excitation energy calculation were carried out at the CAM-B3LYP/6-31G+ (d) level of theory.

The geometry of the complex of ***p*-2Urea** with two equivalents of AcO^- was first optimised at the CAM-B3LYP/6-31G+ (d) level of theory before TD-DFT excitation energy calculations were carried out at the CAM-B3LYP/6-31G+ (d) level.

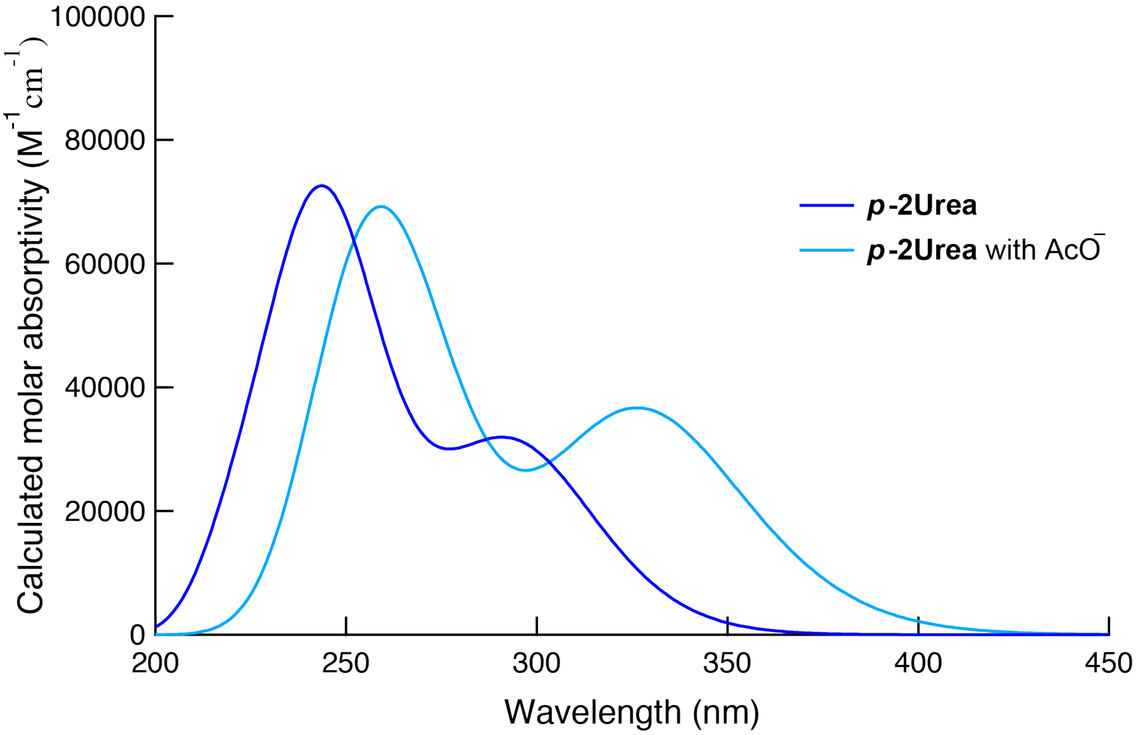

Supplementary Figure 9. Calculated absorption spectrum of ***p*-2Urea** and ***p*-2Urea** with two equivalents of AcO^-.

Supplementary Table 2. Calculated excitation energies and oscillator strengths from the ground state, with orbital configuration contributions, for the ground-state optimised geometry of ***p*-2Urea**.

Excited state	Transition energy / eV	Oscillator strength *f*	Configuration and percentage contribution[a]
S_1	4.193 (295 nm)	0.6383	HOMO→LUMO (92.1%)
S_2	4.352 (285 nm)	0.1230	HOMO−1→LUMO (63.5%)
S_3	4.434 (280 mm)	0.0109	HOMO−1→LUMO (37.4%)
S_4	4.744 (261 nm)	0.0340	HOMO−1→LUMO (54.3%)
S_5	4.805 (258 nm)	0.001	HOMO−1→LUMO+1 (72.8%)

[a]Percentages for the single-particle contributions (y %) to the vertical excited states were calculated using the following expression:

$$y\,\% = \frac{{x_i}^2}{\sum_{i=1}^{n} {x_i}^2} \times 100,$$

where $\boldsymbol{x_i}$ is the single-particle transition corresponding to a given vertical excited state.

Supplementary Table 3. Calculated excitation energies and oscillator strengths from the ground-state, with orbital configuration contributions, for the ground-state optimised geometry of ***p*-2Urea** with two equivalents of AcO^-.

Excited state	Transition energy / eV	Oscillator strength *f*	Configuration and percentage contribution[a]
S_1	3.704 (335 nm)	0.2356	HOMO→LUMO (77.0%)
S_2	3.805 (326 nm)	0.6503	HOMO−1→LUMO (71.2%)
S_3	4.237 (293 nm)	0.0776	HOMO−1→LUMO+3 (38.7%)
S_4	4.613 (269 nm)	0.3360	HOMO→LUMO+3 (67.7%)
S_5	4.660 (266 nm)	0.1118	HOMO→LUMO+8 (16.6%)

[a]The percentages were calculated using the expression given in the footnote of Supplementary Table 2.

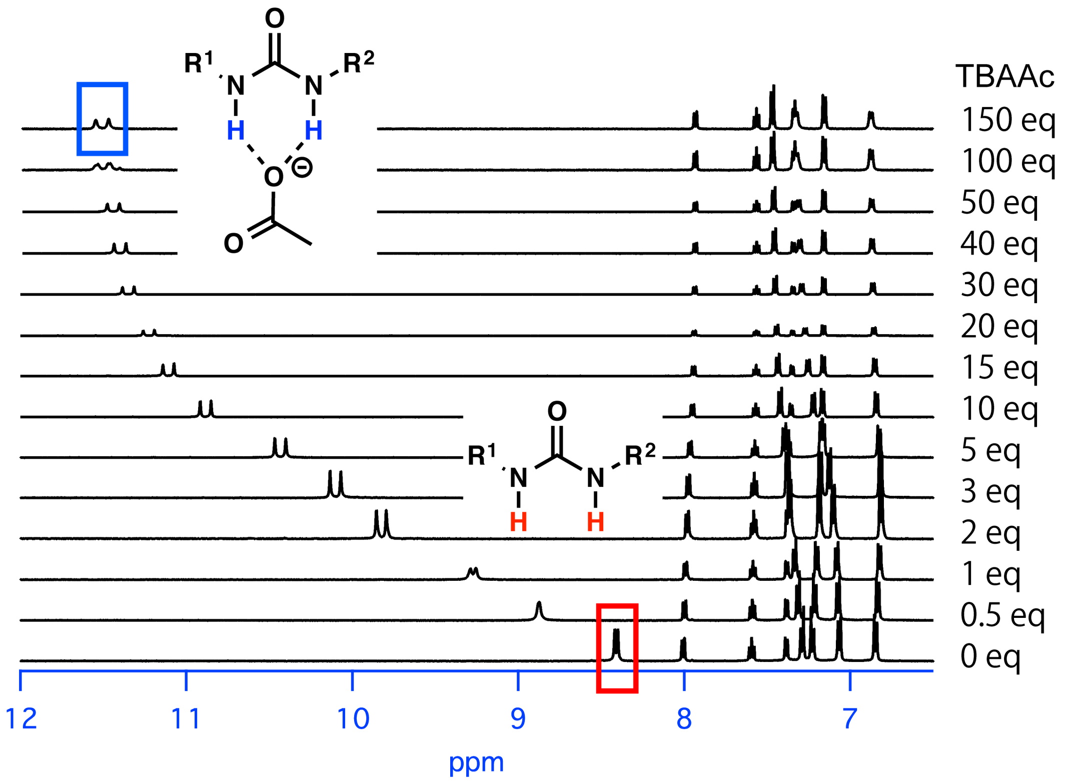

Supplementary Figure 10. ^{1}H NMR spectra acquired over the course of titrating a solution of ***p*-2Urea** (2.0 mM) with tetrabutylammonium acetate (TBAAc) in DMSO-d_6.

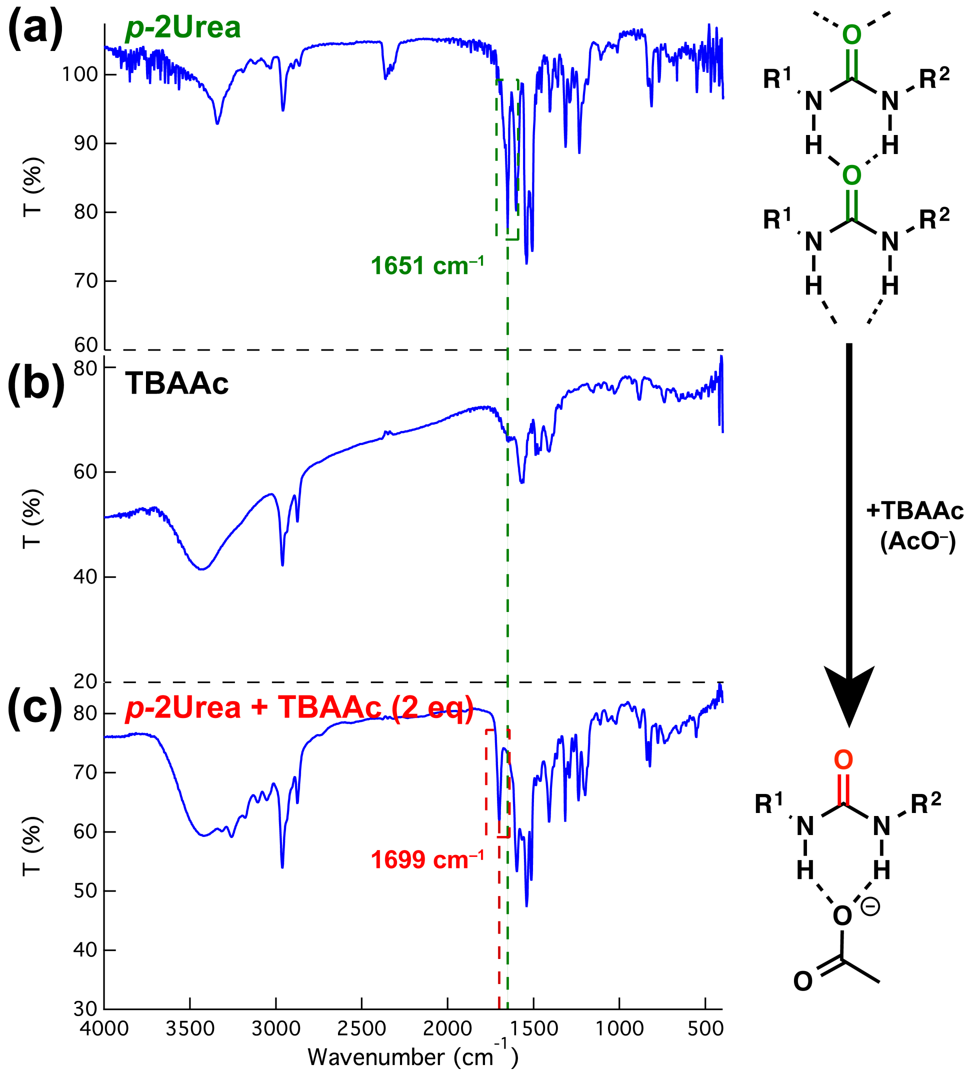

Supplementary Figure 11. FT-IR spectra of ***p*-2Urea** and ***p*-2Urea**–acetate-ion complex in solid-state. (a) FT-IR spectra of TBAAc. (b) FT-IR spectra of ***p*-2Urea**. (c) FT-IR spectra of the mixture of ***p*-2Urea** and TBAAc.

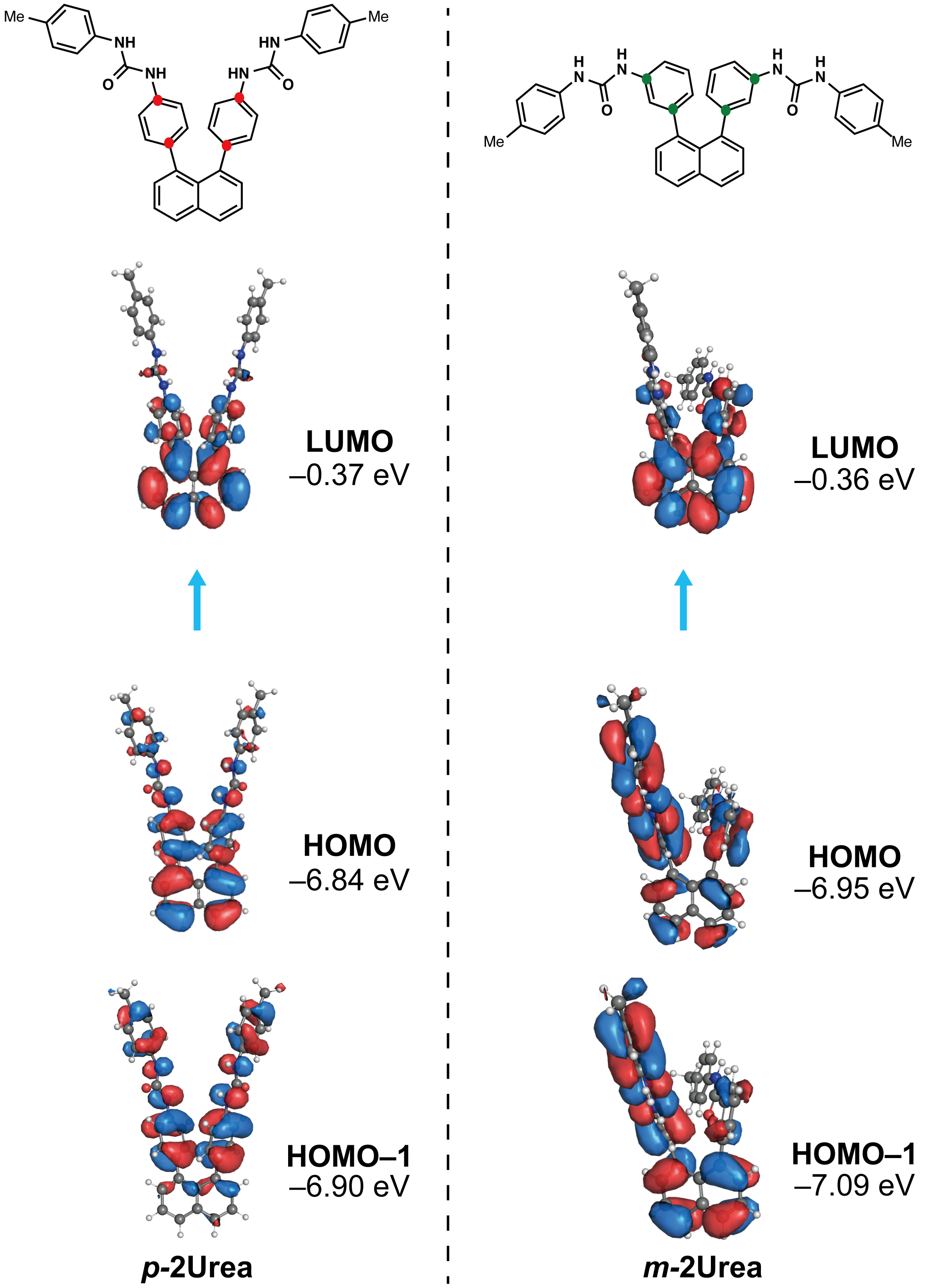

Supplementary Figure 12. Frontier molecular orbital amplitude plots of the HOMOs and LUMOs of ***p*-2Urea** (left) and ***m*-2Urea** (right). The geometry was first optimised at the B3LYP/6-31G (d, p) level of theory before TD-DFT excitation energy calculations were carried out at the CAM-B3LYP/6-31G+ (d) level.

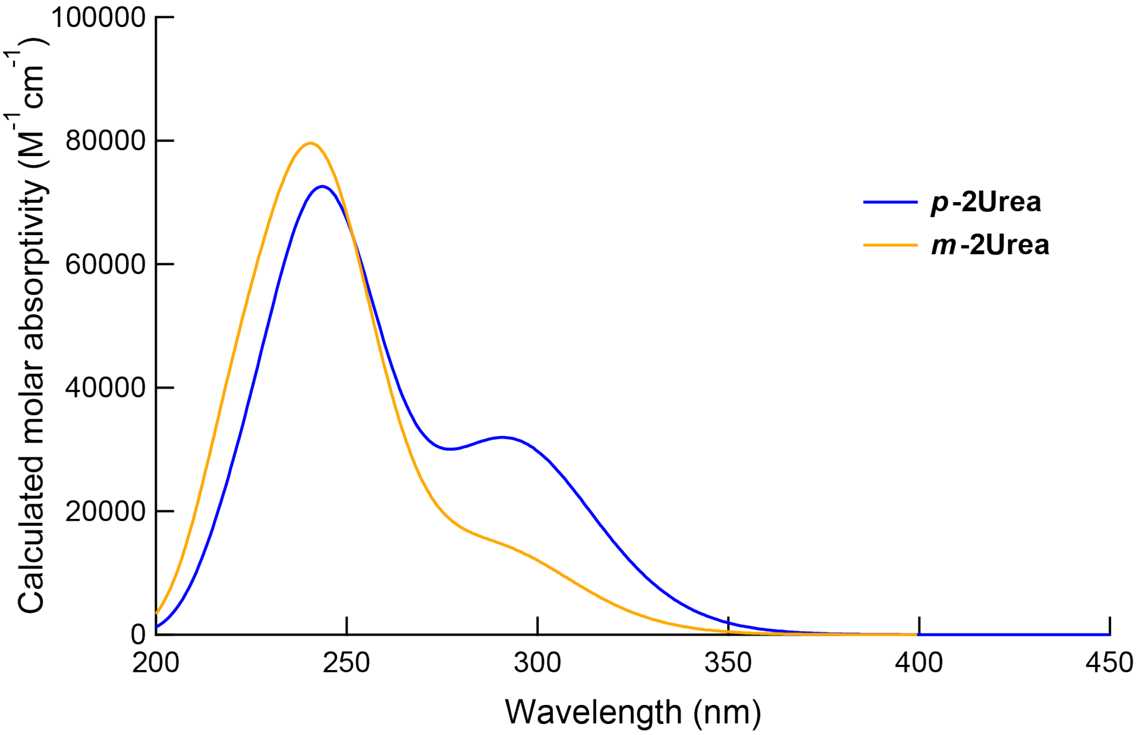

Supplementary Figure 13. Calculated absorption spectra of ***p*-2Urea** and ***m*-2Urea**.

Supplementary Table 4. Calculated excitation energies and oscillator strengths from the ground state, with orbital configuration contributions, for the ground-state optimised geometry of ***m*-2Urea**.

Excited state	Transition energy / eV	Oscillator strength *f*	Configuration and percentage contribution[a]
S_1	4.267 (291 nm)	0.3153	HOMO→LUMO (54.8%)
S_2	4.427 (280 nm)	0.0056	HOMO−6→LUMO (36.1%)
S_3	4.662 (266 nm)	0.0061	HOMO→LUMO (31.6%)
S_4	4.779 (259 nm)	0.0338	HOMO→LUMO+4 (37.3%)
S_5	4.819 (257 nm)	0.0092	HOMO−1→LUMO+2 (26.0%)

[a]The percentages were calculated using the expression given in the footnote of Supplementary Table 2.

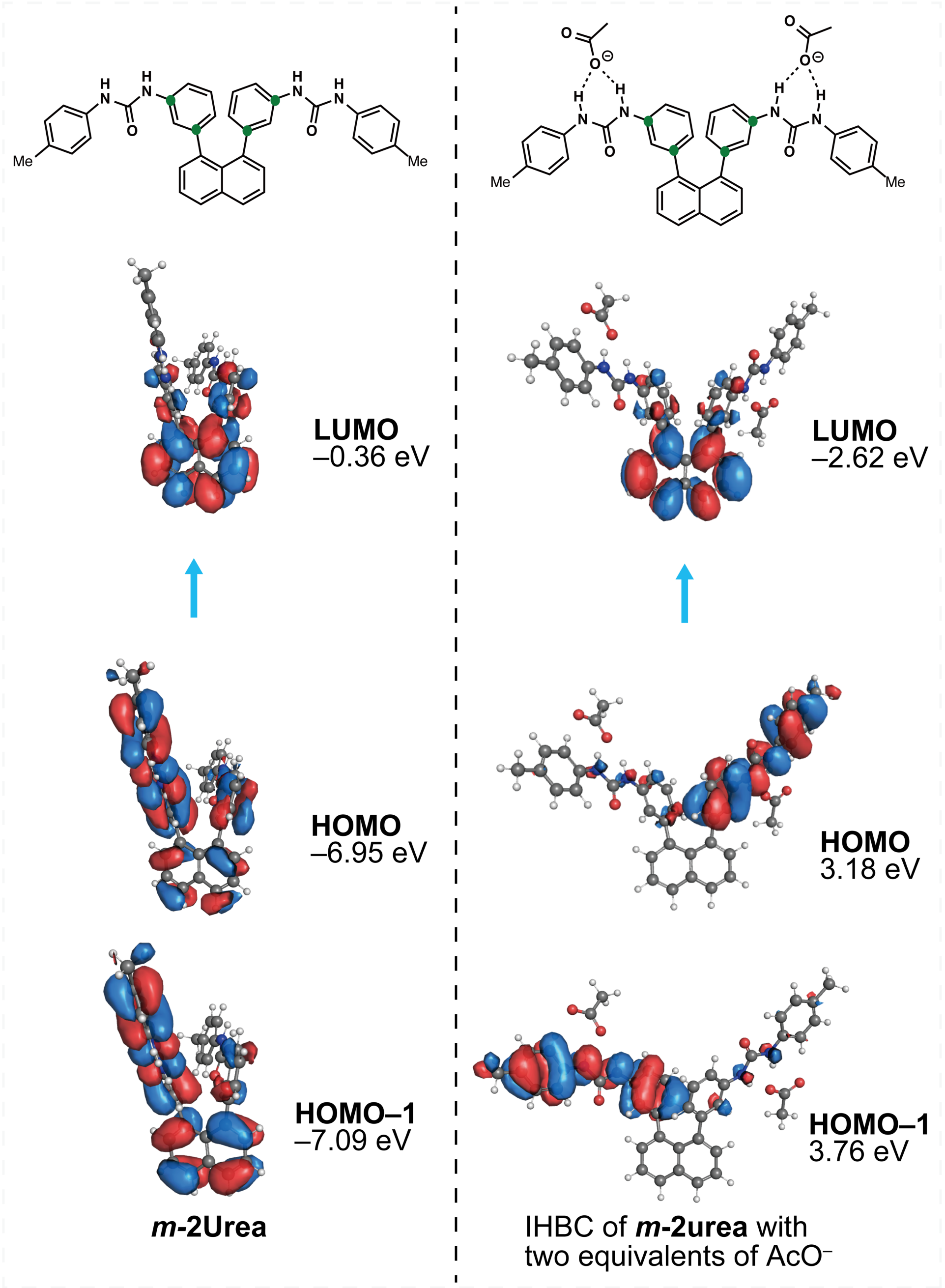

Supplementary Figure 14. Frontier molecular orbital amplitude plots of the HOMO and LUMO of ***m*-2Urea** (left) and the complex of ***m*-2Urea** with two equivalents of AcO^- (right). The geometry of ***m*-2Urea** was first optimised at the B3LYP/6-31G (d, p) level of theory before TD-DFT excitation energy calculations were carried out at the CAM-B3LYP/6-31G+ (d) level. The geometry of the complex of ***m*-2Urea** with two equivalents of AcO^- was first optimised at the CAM-B3LYP/6-31G+ (d) level of theory before TD-DFT excitation energy calculations were carried out at the CAM-B3LYP/6-31G+ (d) level.

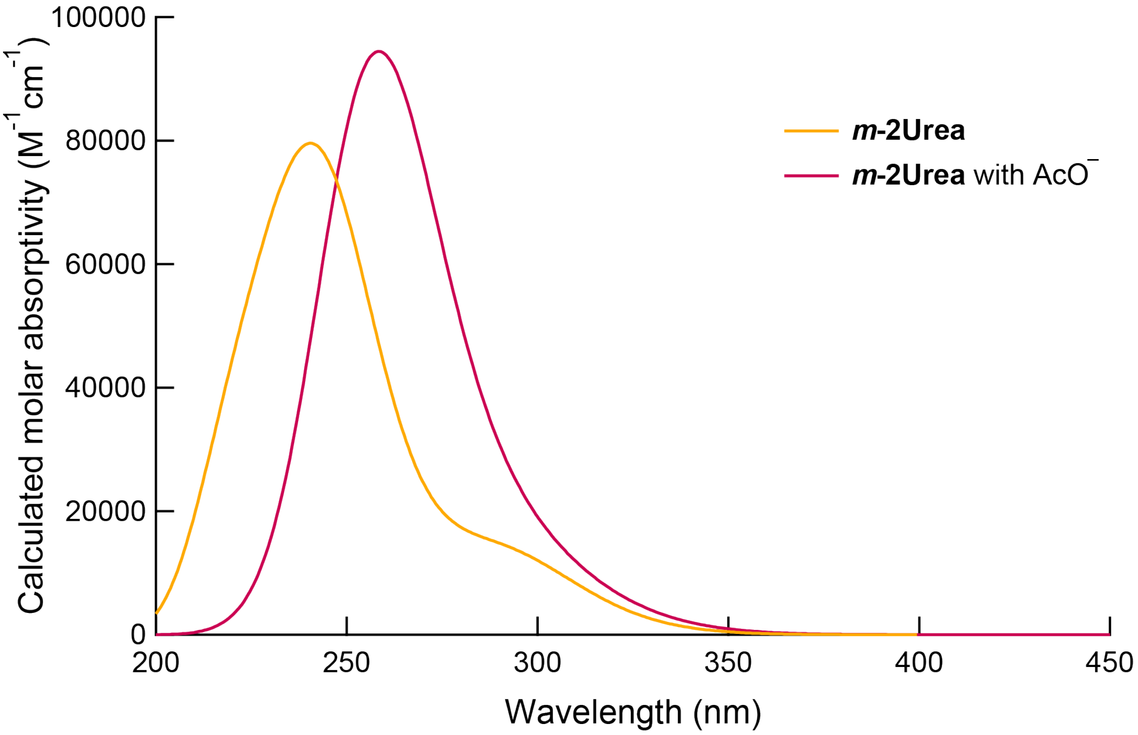

Supplementary Figure 15. Calculated absorption spectra of ***m*-2Urea** and ***m*-2Urea** with two equivalents of AcO^-.

Supplementary Table 5. Calculated excitation energies and oscillator strengths from the ground state, with orbital configuration contributions, for the ground-state optimised geometry of the complex ***m*-2Urea** and two equivalents of AcO^-

Excited state	Transition energy / eV	Oscillator strength *f*	Configuration and percentage contribution[a]
S_1	4.054 (306 nm)	0.0173	HOMO→LUMO (54.7%)
S_2	4.120 (301 nm)	0.1214	HOMO−1→LUMO (49.0%)
S_3	4.328 (286 nm)	0.2277	HOMO−6→LUMO (80.7%)
S_4	4.458 (278 nm)	0.0300	HOMO−6→LUMO+4 (34.4%)
S_5	4.761 (260 nm)	0.1138	HOMO−11→LUMO (52.4%)

[a]The percentages were calculated using the expression given in the footnote of Supplementary Table 2.

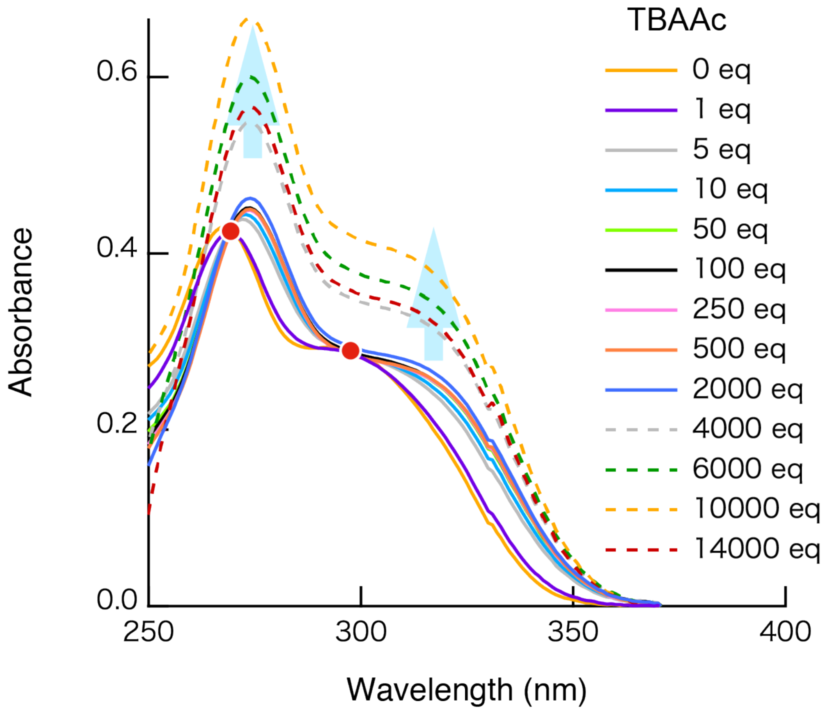

Supplementary Figure 16. Absorbance spectra of ***p*-1Urea** in the absence and presence of TBAAc.

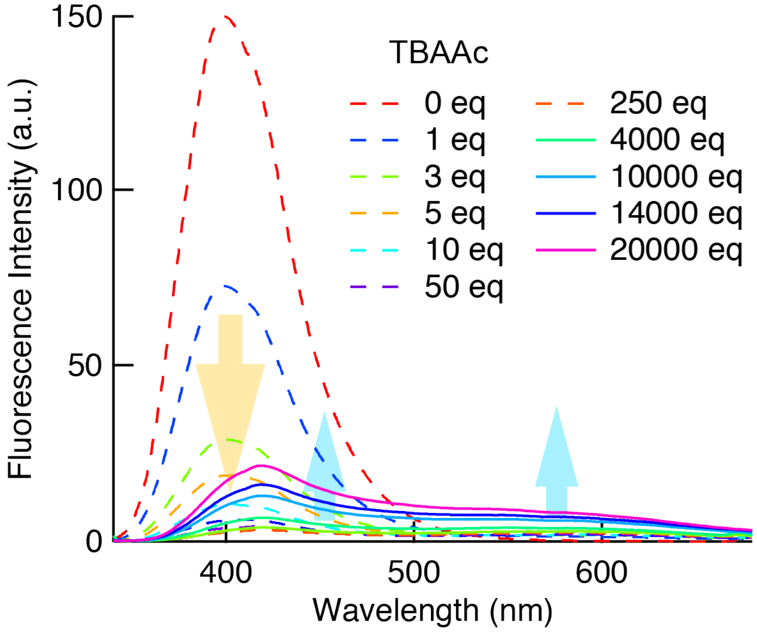

Supplementary Figure 17. Fluorescence spectra of a DMSO solution of ***p*-1Urea** (150 μM, λ_{ex} = 336 nm) in the absence and presence of TBAAc.

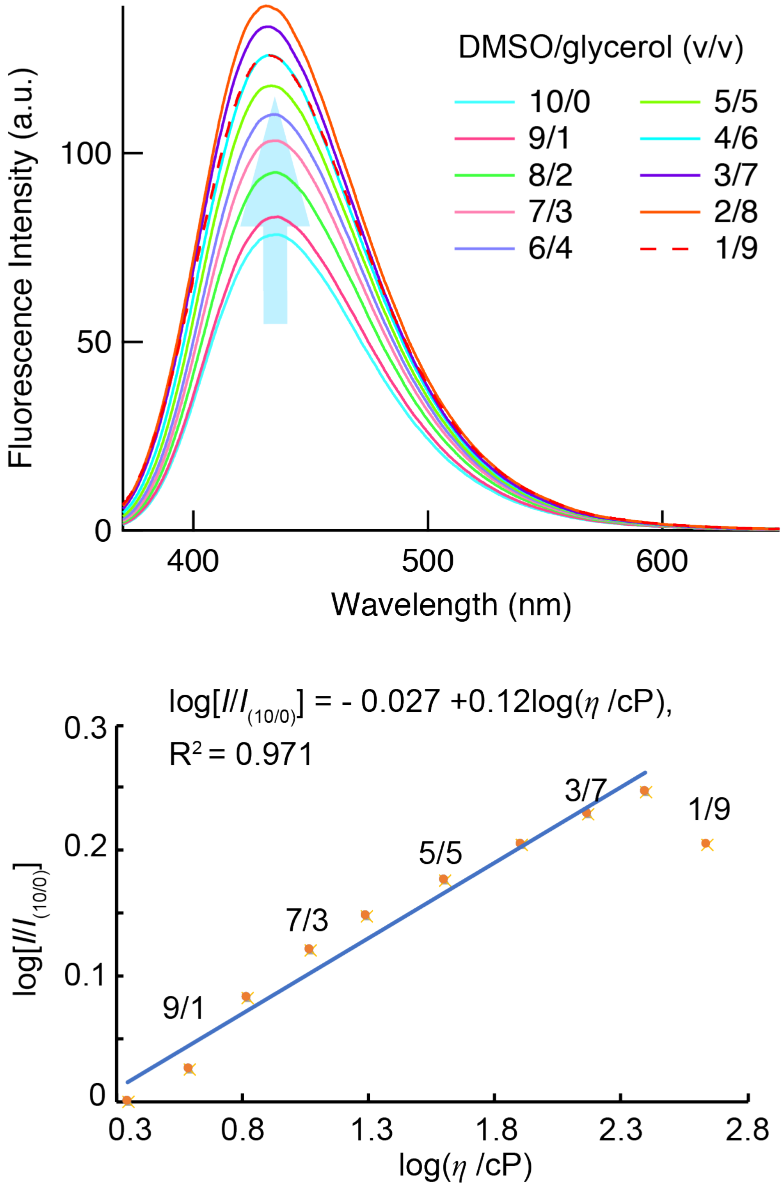

Supplementary Figure 18. Viscosity-sensitivity of the ***p*-2Urea** and fluorescence intensity. (a) Fluorescence spectra of ***p*-2Urea** in mixed solvents with different fractions of glycerol [DMSO/glycerol (v/v) = 10/0, 9/1, 8/2, 7/3, 6/4, 5/5, 4/6, 3/7, 2/8, and 1/9]. (b) Linear relationship between log($I/I_{(v/v)}$) and log(η /cP) in mixtures of DMSO and glycerol ([***p*-2Urea**] = 1.5 μM, λ_{ex} = 359 nm).

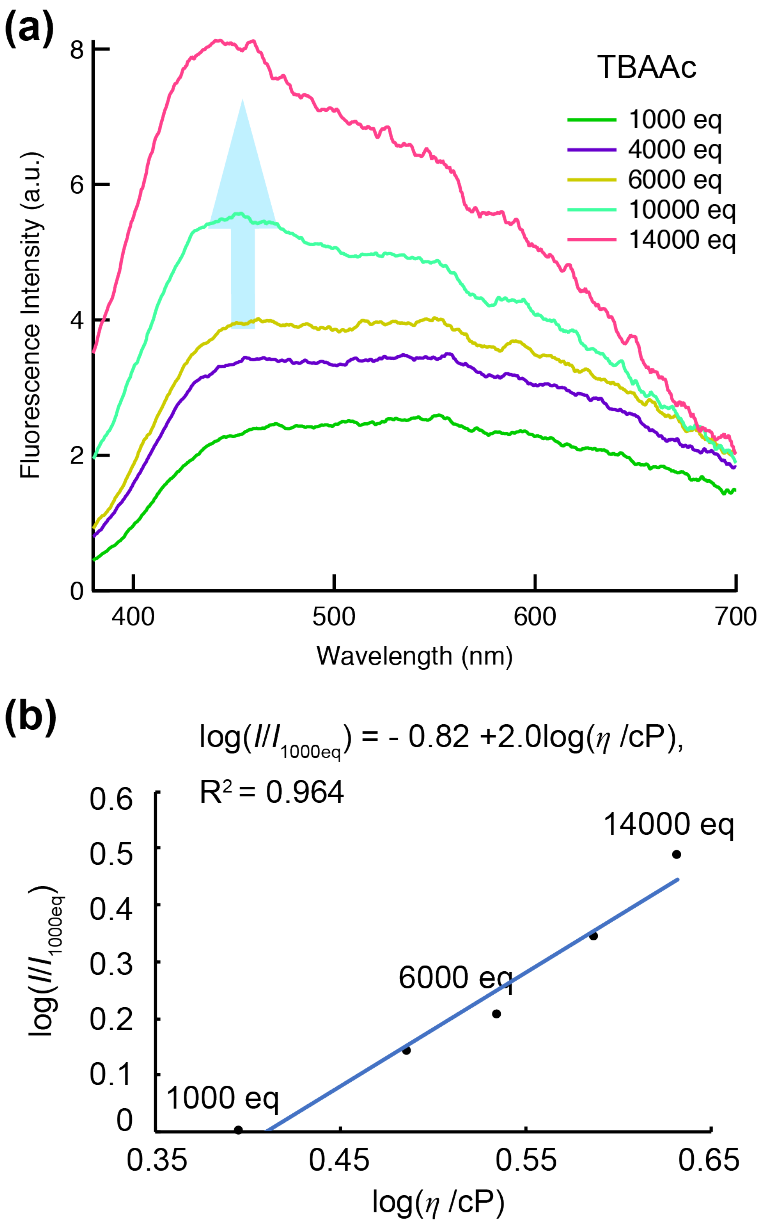

Supplementary Figure 19. Viscosity-sensitivity of the ***m*-2Urea** complex and fluorescence intensity. (a) Fluorescence spectra of ***m*-2Urea** (150 µM, λ_{ex}= 332 nm) with the addition of various excess amounts of TBAAc in DMSO. (b) Log–log plot of the ratio of fluorescence intensities at 470.5 nm as a function of solvent viscosity.

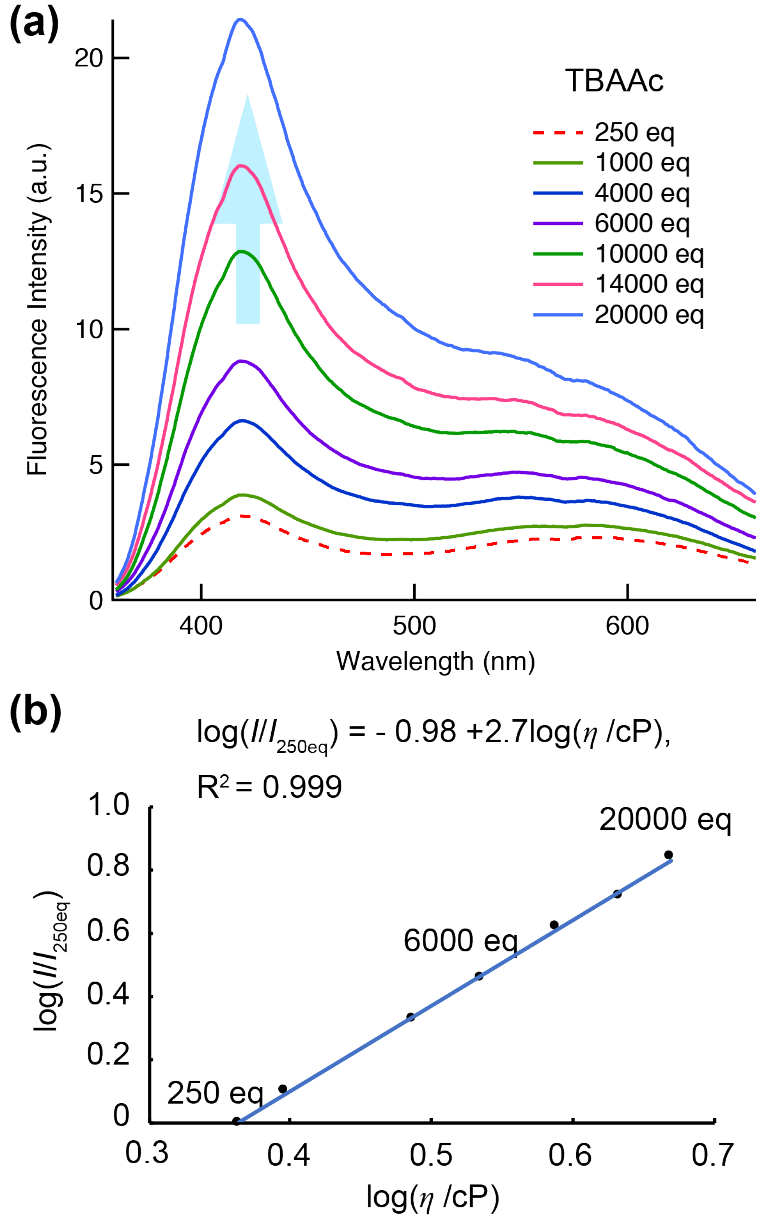

Supplementary Figure 20. Viscosity-sensitivity of the ***p*-1Urea** complex and fluorescence intensity. (a) Fluorescence spectra of ***p*-1Urea** (150 µM, λ_{ex}= 336 nm) with the addition of various excess amounts of TBAAc in DMSO. (b) Log–log plot of the ratio of fluorescence intensities at 418 nm as a function of solvent viscosity.

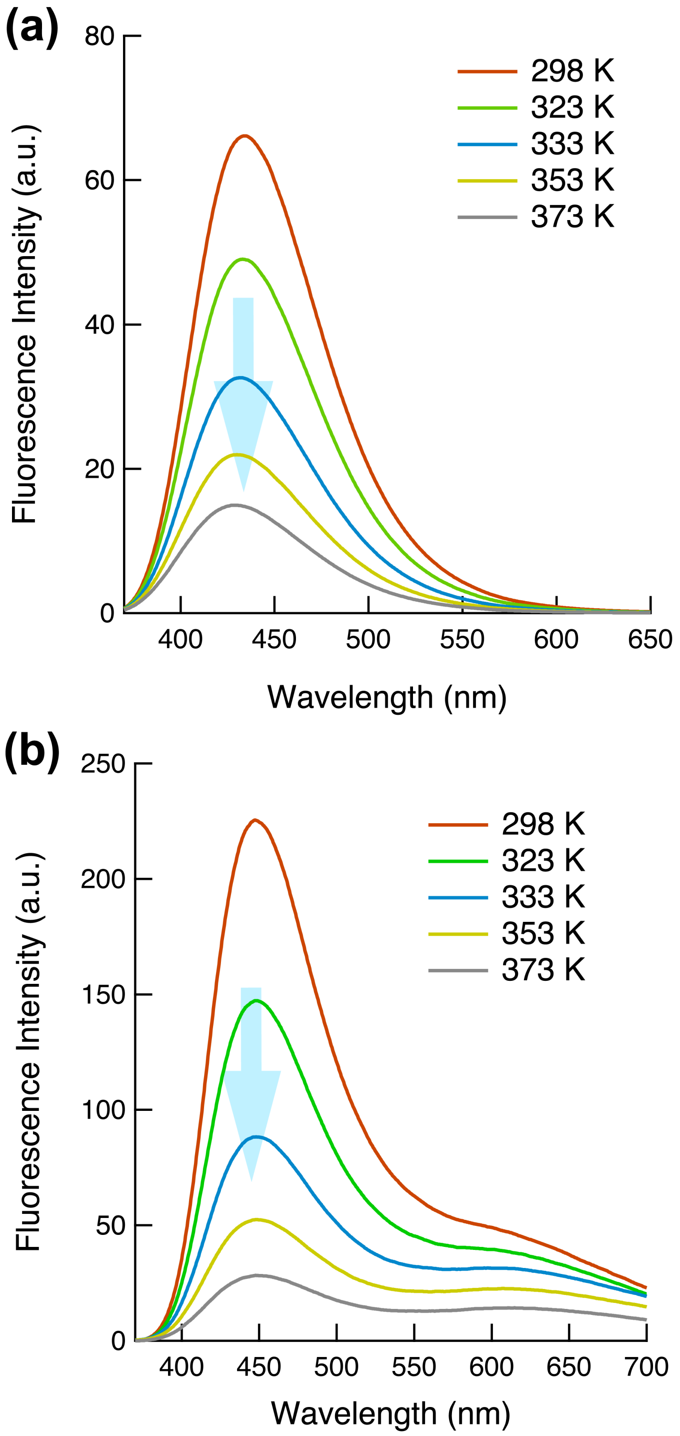

Supplementary Figure 21. Temperature dependences of fluorescence emissions of the ***p*-2Urea** in the absence and presence of TBAAc. (a) Fluorescence spectra of ***p*-2Urea** (150 μM, λ_{ex}= 336 nm) in DMSO at different temperatures (298 K to 373 K). (b) Fluorescence spectra of ***p*-2Urea** (150 μM, λ_{ex}= 336 nm) with the addition of 14000 equivalents of TBAAc in DMSO at different temperatures (298 K to 373 K).

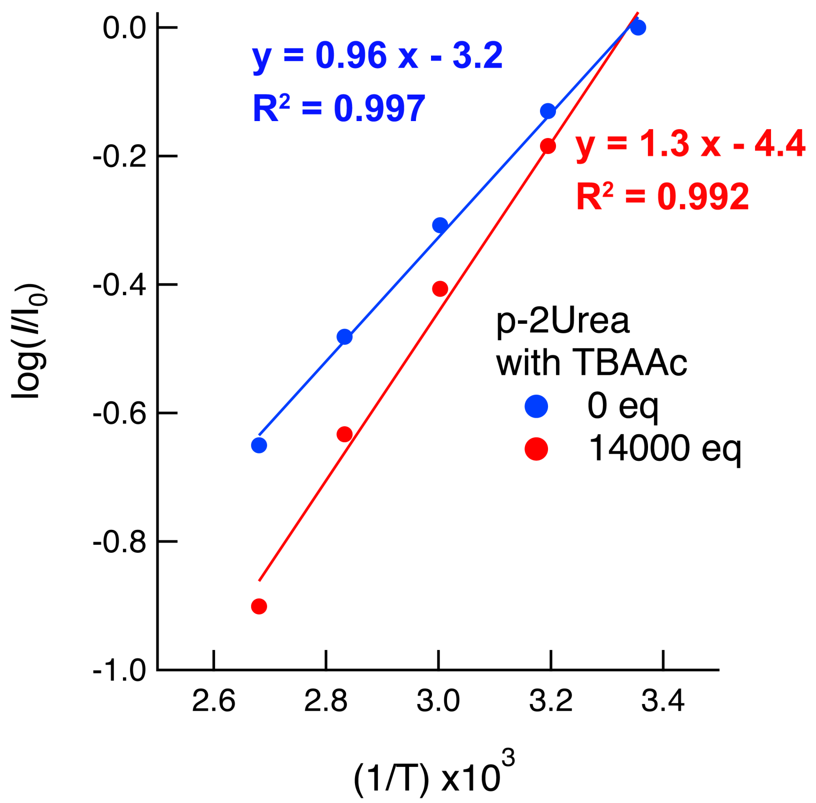

Supplementary Figure 22. Temperature dependences of fluorescence emissions of the ***p*-2Urea** in the absence and presence of TBAAc. Fluorescence intensity changes of ***p*-2Urea** (150 µM, λ_{ex}= 336 nm, λ_{em}= 448 nm) with the addition of 14000 equivalents of TBAAc in DMSO and ***p*-2Urea** (150 µM, λ_{ex}= 336 nm, λ_{em}= 434 nm) without TBAAc in DMSO at different temperatures (298 K to 373 K).

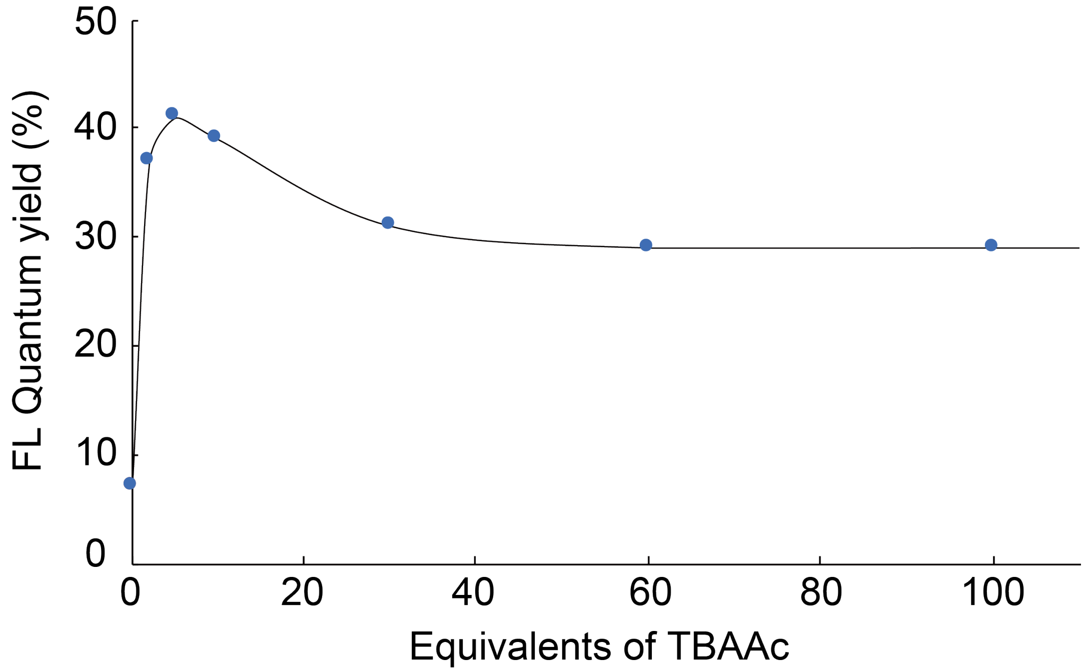

Supplementary Figure 23. Fluorescence quantum yields of ***p*-2Urea** powders in the presence of various amounts of TBAAc and in its absence.

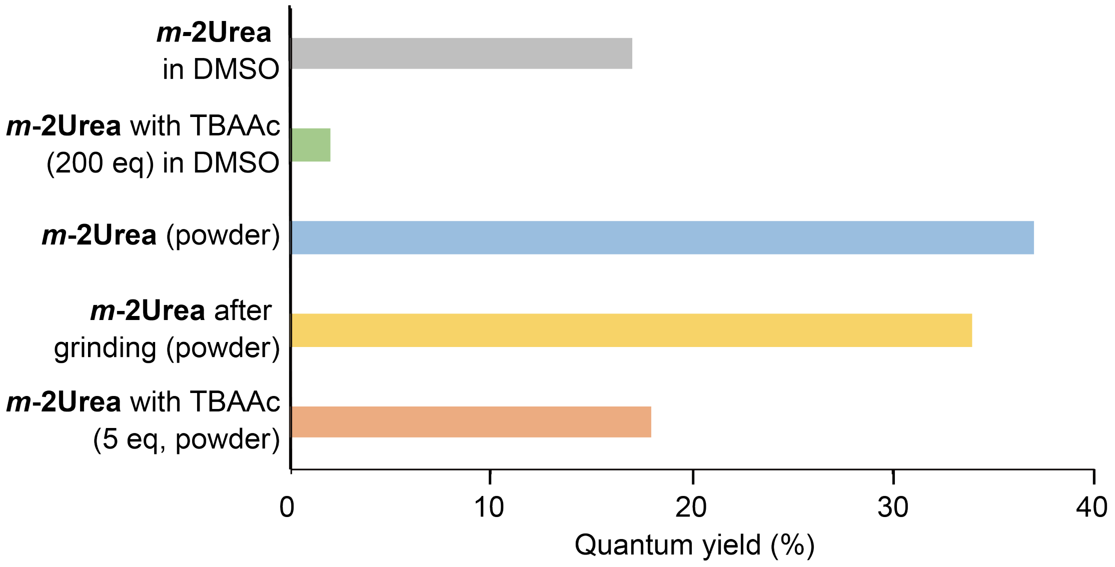

Supplementary Figure 24. Absolute fluorescence quantum yields of ***m*-2Urea** powder and ***m*-2Urea**–DMSO solution in the absence and presence of TBAAc.

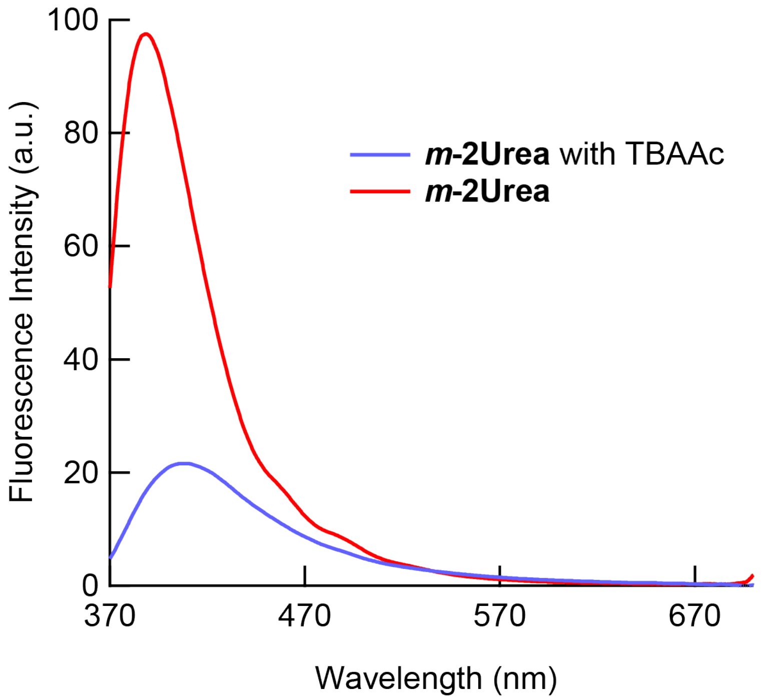

Supplementary Figure 25. Solid-state fluorescence spectra of ***m*-2Urea** in the presence and absence of 5 equiv of TBAAc when excited at 323 nm.

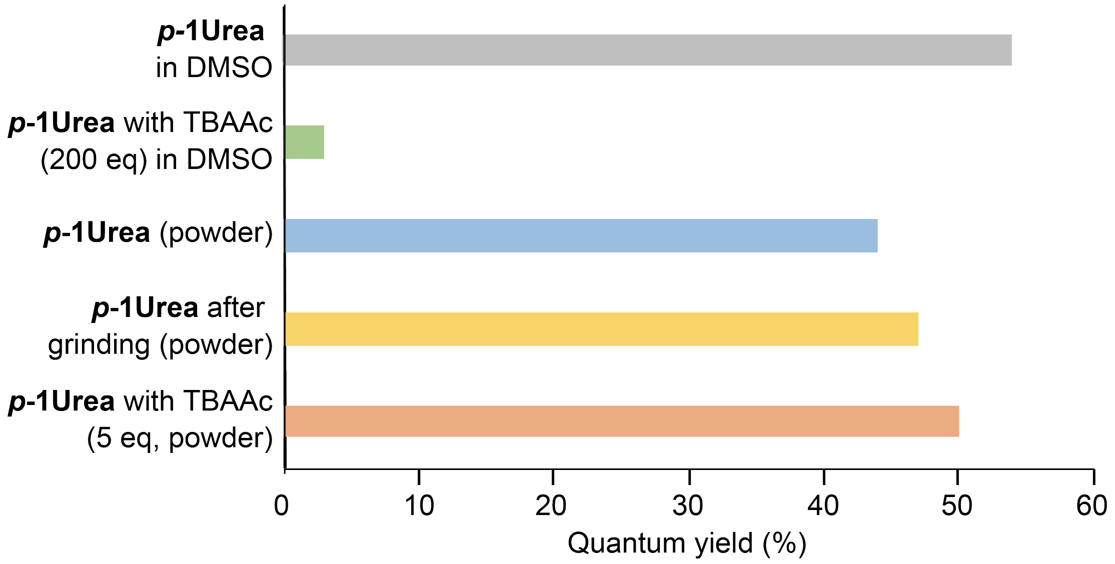

Supplementary Figure 26. Absolute fluorescence quantum yields of ***p*-1Urea** powder and ***p*-1Urea**–DMSO solution in the absence and presence of TBAAc.

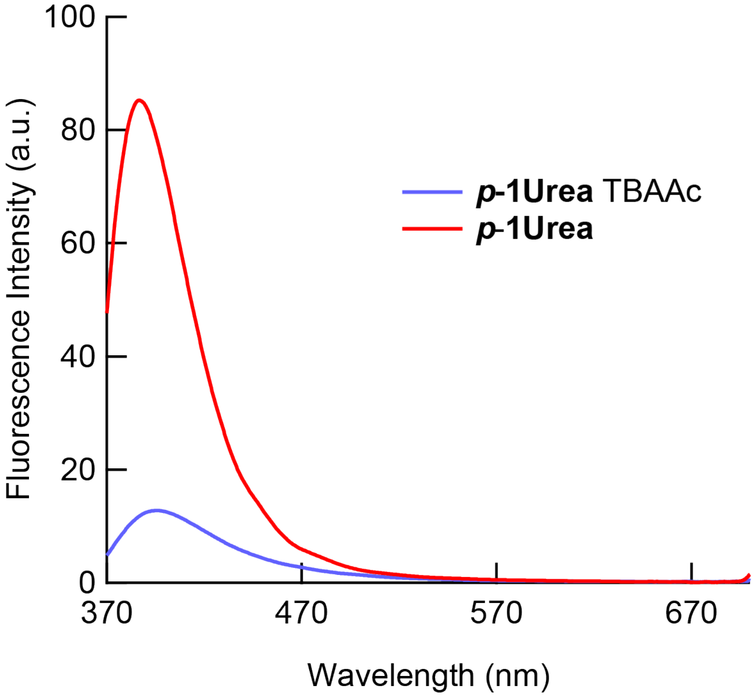

Supplementary Figure 27. Solid-state fluorescence spectra of ***p*-1Urea** in the presence and absence of 5 equiv of TBAAc when excited at 323 nm.

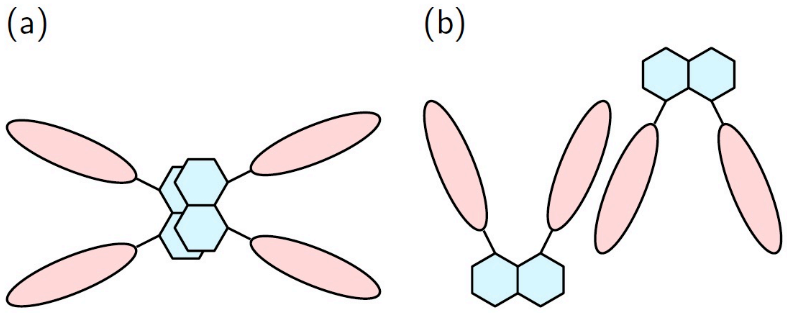

Supplementary Figure 28. A schematic illustration of dimer models of ***p*-2Urea**: (a) type (i): a naphthalene-naphthalene stacking model and (b) type (ii): a urea-urea stacking model.

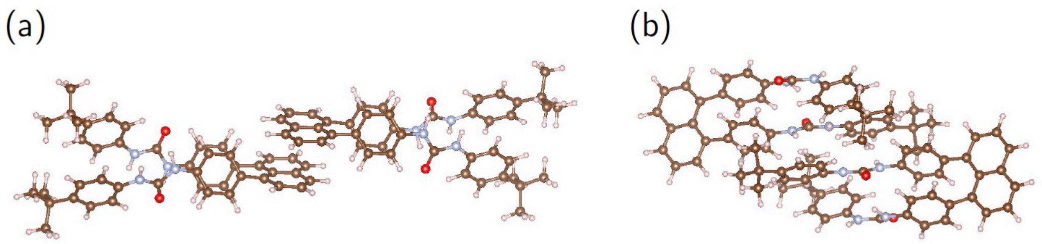

Supplementary Figure 29. S_0-optimised structures of the ***p*-2Urea** dimers in the absence of acetate ions: (a) type (i) and (b) type (ii) with C_i symmetry at the B3LYP/3-21G level of theory with the Grimme's empirical dispersion D3.

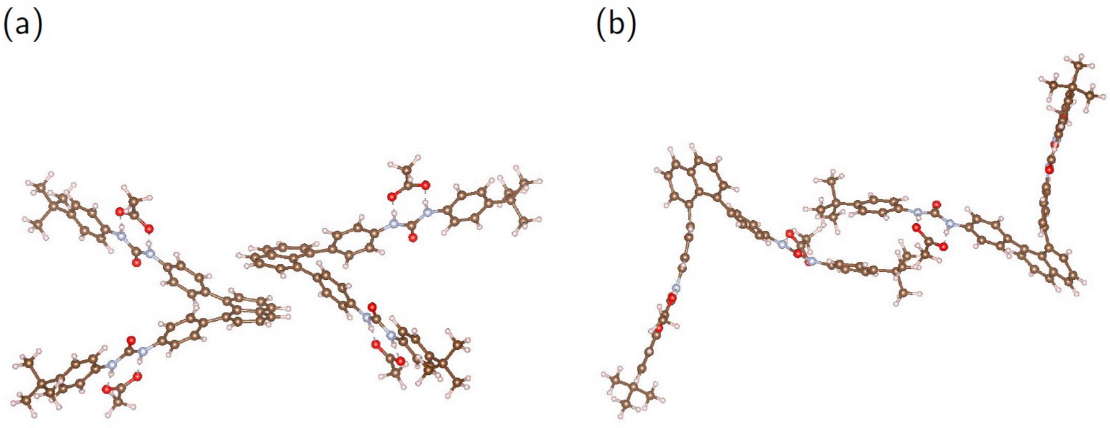

Supplementary Figure 30. S_0-optimised structures of the ***p*-2Urea** dimers in the presence of acetate ions: (a) type (i) and (b) type (ii) with C_i symmetry at the B3LYP/3-21G level of theory with the Grimme's empirical dispersion D3.

Supplementary Table 6. Excited States of the ***p*-2Urea** dimer (ii) in the absence of acetate ions at the S_0-optimised structure at the B3LYP/3-21G level of theory with the Grimme's empirical dispersion D3.

state	excitation energy eV	nm	oscillator strength f	major configurations
S_1 (^1A_u)	3.6411	340.51	0.0556	351 → 354 (0.45691)
				352 → 353 (0.51873)
S_2 (^1A_g)	3.6416	340.46	0.0000	351 → 353 (0.46438)
				352 → 354 (0.51224)
S_3 (^1A_g)	4.0210	308.34	0.0000	351 → 353 (0.52117)
				352 → 354 (-0.46400)
S_4 (^1A_u)	4.0212	308.32	0.0469	351 → 354 (0.52801)
				352 → 353 (-0.45478)
S_5 (^1A_u)	4.1597	298.06	0.7588	349 → 354 (0.37333)
				350 → 353 (0.56107)
S_6 (^1A_g)	4.1734	297.08	0.0000	349 → 353 (0.38333)
				350 → 354 (0.55289)
S_7 (^1A_g)	4.3174	287.17	0.0000	348 → 353 (0.30738)
				351 → 355 (0.32682)
				352 → 356 (0.47695)
S_8 (^1A_u)	4.3205	286.97	0.0532	351 → 356 (0.36946)
				352 → 355 (0.50004)
S_9 (^1A_g)	4.3418	285.56	0.0000	348 → 353 (0.44603)
				349 → 353 (-0.40202)
S_{10} (^1A_u)	4.3479	285.16	0.0078	348 → 354 (0.46160)
				349 → 354 (-0.44133)

Supplementary Table 7. Excited States of the ***p*-2Urea** dimer (ii) in the presence of acetate ions at the S_0-optimised structure at the B3LYP/3-21G level of theory with the Grimme's empirical dispersion D3.

state	excitation energy eV	nm	oscillator strength f	major configurations
S_1 (^1A)	2.2364	554.40	0.2144	416 → 417 (0.66739)
S_2 (^1A)	2.2874	542.04	0.1187	415 → 418 (0.52146)
				416 → 418 (-0.45654)
S_3 (^1A)	2.3760	521.81	0.0155	415 → 418 (0.45687)
				416 → 418 (0.53598)
S_4 (^1A)	2.4196	512.41	0.0037	415 → 417 (0.67786)
S_5 (^1A)	2.7660	448.24	0.0759	414 → 417 (0.66662)
S_6 (^1A)	2.8024	442.43	0.0678	413 → 418 (0.65616)
S_7 (^1A)	2.9772	416.45	0.1813	411 → 417 (0.57869)
				416 → 419 (0.34514)
S_8 (^1A)	2.9979	413.56	0.2315	412 → 418 (0.63135)
S_9 (^1A)	3.0148	411.25	0.0022	414 → 418 (0.67726)
S_{10} (^1A)	3.0287	409.37	0.0001	413 → 417 (0.69088)

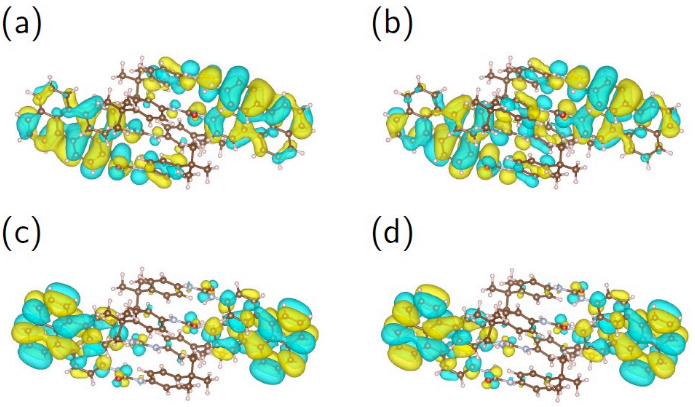

Supplementary Figure 31. Frontier orbitals of the ***p*-2Urea** dimer (ii) in the absence of acetate ions at the S_0-optimised structure at the B3LYP/3-21G level of theory with the Grimme's empirical dispersion D3: (a) NHOMO, (b) HOMO, (c) LUMO, and (d) NLUMO. The isosurface value is 1.0×10^{-2} a.u.

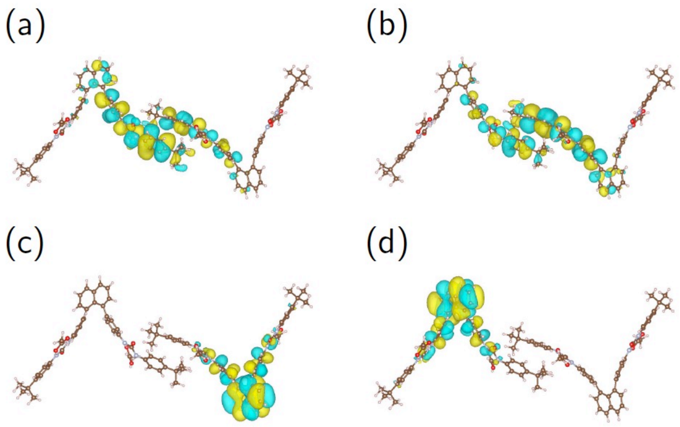

Supplementary Figure 32. Frontier orbitals of the ***p*-2Urea** dimer (ii) in the presence of acetate ions at the S_0-optimised structure at the B3LYP/3-21G level of theory with the Grimme's empirical dispersion D3: (a) NHOMO, (b) HOMO, (c) LUMO, and (d) NLUMO. The isosurface value is 1.0×10^{-2} a.u.

3. Supplementary References

1. Ghosn, M. W. et al. Stereocontrolled Photodimerization with Congested 1, 8-Bis (4′-anilino) naphthalene Templates. *J Org Chem.* **75**, 6653-6659 (2010).
2. Takahashi, M. et al. A novel approach to white-light emission using a single fluorescent urea derivative and fluoride. *New Journal of Chemistry.* **43**, 3265-3268 (2019).
3. Frisch, M. J. et al. Gaussian 16 Rev. C.01. Wallingford, CT: Gaussian, Inc.; 2016.
4. Yanai, T. et al. A new hybrid exchange–correlation functional using the Coulomb-attenuating method (CAM-B3LYP). *Chem Phys Lett.* **393**, 51-57 (2004).
5. Hariharan, P. C. et al. The influence of polarization functions on molecular orbital hydrogenation energies. *Theo Chim Acta.* **28**, 213-222 (1973).
