# Supplementary material for: Environment-sensitive emission of anionic hydrogen-bonded urea-derivative–acetate-ion complexes and their aggregation-induced emission enhancement
Source: Commun Chem. 2021 Dec 2;4:168. doi: 10.1038/s42004-021-00601-3 (PMC9814938; doi:10.1038/s42004-021-00601-3)
Supplement: Supplementary file 3 — Supplementary Data 2 [file 42004_2021_601_MOESM3_ESM.docx]

Environment-sensitive emission of anionic hydrogen-bonded urea-derivative–acetate-ion complexes and their aggregation-induced emission enhancement

Supplementary Data

Masaki Takahashi*, Nozomu Ito, Naoki Haruta, Hayato Ninagawa, Kohei Yazaki, Yoshihisa Sei, Tohru Sato, and Makoto Obata

**Supplementary Data 2.** Structure of ***p*-2Urea**, S_0_ [DFT/B3LYP/6-31G (d, p), *E*_sp_ = -1836.90938116 hartree].

| atom | x[Å] | y[Å] | z[Å] |
| --- | --- | --- | --- |
| C | -7.70735 | -4.82948 | 0.69874 |
| H | -7.03939 | -3.4336 | -1.56866 |
| C | -6.35664 | -4.29338 | 0.28748 |
| C | -6.18357 | -3.60848 | -0.92147 |
| C | -5.22278 | -4.48247 | 1.08574 |
| H | -5.32238 | -5.0058 | 2.03377 |
| C | -4.93405 | -3.14058 | -1.31401 |
| H | -4.83326 | -2.60649 | -2.25683 |
| C | -3.9602 | -4.02387 | 0.71248 |
| C | -3.80607 | -3.34317 | -0.50407 |
| H | -3.10088 | -4.18111 | 1.34766 |
| H | -2.62942 | -2.40704 | -1.88753 |
| N | -2.57544 | -2.84056 | -0.9776 |
| C | -1.34027 | -2.86159 | -0.34656 |
| O | -1.14645 | -3.34512 | 0.76153 |
| H | -0.62448 | -1.85281 | -1.99405 |
| N | -0.3479 | -2.27559 | -1.12004 |
| H | 1.04188 | -3.18386 | 1.05419 |
| C | 1.00514 | -2.06549 | -0.7839 |
| C | 1.61847 | -2.58459 | 0.365 |
| H | -2.65939 | 2.32257 | 1.84522 |
| H | -4.86987 | 2.4879 | 2.19089 |
| H | -0.6449 | 1.8116 | 1.9728 |
| C | 1.77216 | -1.29561 | -1.67341 |
| H | 1.31397 | -0.8987 | -2.57688 |
| C | 2.96074 | -2.30225 | 0.61013 |
| H | 1.30062 | 0.88135 | 2.56699 |
| C | -4.96022 | 3.07185 | 1.27703 |
| H | 3.41791 | -2.69679 | 1.51328 |
| H | -7.07122 | 3.33763 | 1.51477 |
| C | 1.75917 | 1.28238 | 1.66555 |
| N | -0.36598 | 2.2462 | 1.10538 |
| N | -2.59442 | 2.80707 | 0.96211 |
| C | -6.207 | 3.55275 | 0.8913 |
| H | 3.66757 | 0.42628 | 2.11427 |
| C | 3.09739 | 1.02018 | 1.40758 |
| C | 3.10804 | -1.025 | -1.41171 |
| C | 0.99024 | 2.04917 | 0.7749 |
| C | -3.82173 | 3.3251 | 0.4967 |
| C | 3.73039 | -1.50698 | -0.25069 |
| C | -1.35326 | 2.85629 | 0.34435 |
| C | 3.72057 | 1.50782 | 0.24935 |
| C | 1.6041 | 2.5739 | -0.37121 |
| C | -6.36666 | 4.30126 | -0.28114 |
| H | 3.67687 | -0.42865 | -2.11744 |
| O | -1.15104 | 3.38097 | -0.74332 |
| C | -3.9623 | 4.07015 | -0.68329 |
| C | 2.94886 | 2.30002 | -0.61245 |
| C | 5.18239 | -1.28301 | 0.0071 |
| C | 5.17459 | 1.29282 | -0.00429 |
| C | -7.71465 | 4.85061 | -0.68376 |
| H | 1.02635 | 3.17218 | -1.06027 |
| C | -5.22225 | 4.54052 | -1.0505 |
| H | 5.4405 | -3.39047 | 0.16047 |
| C | 5.8367 | 0.00689 | 0.00232 |
| H | 5.42056 | 3.40179 | -0.15679 |
| C | 5.93347 | 2.445 | -0.16358 |
| C | 5.94767 | -2.43063 | 0.16856 |
| H | -3.0949 | 4.26601 | -1.29639 |
| H | 3.40646 | 2.69888 | -1.5135 |
| C | 7.33831 | 2.42297 | -0.25897 |
| C | 7.28135 | 0.01122 | 0.00434 |
| C | 7.35209 | -2.40018 | 0.26785 |
| H | -5.31129 | 5.11384 | -1.97025 |
| C | 7.99965 | 1.22724 | -0.13964 |
| H | 7.88659 | 3.35283 | -0.37712 |
| C | 8.00653 | -1.20048 | 0.15034 |
| H | 7.90562 | -3.32671 | 0.3876 |
| H | 9.08552 | 1.18742 | -0.15155 |
| H | 9.0921 | -1.15412 | 0.16533 |
| H | -8.52055 | -4.2084 | 0.31045 |
| H | -7.80633 | -4.87086 | 1.7877 |
| H | -7.86786 | -5.84703 | 0.32012 |
| H | -8.52983 | 4.21122 | -0.33105 |
| H | -7.80223 | 4.9381 | -1.77101 |
| H | -7.88248 | 5.85072 | -0.26404 |

**Supplementary Data 3.** Structure of ***m*-2Urea***,* S_0_ [DFT/B3LYP/6-31G (d, p), *E*_sp_ = -1836.90833149 hartree].

| atom | x[Å] | y[Å] | z[Å] |
| --- | --- | --- | --- |
| C | -1.62002 | -3.20337 | 2.54228 |
| C | -2.15578 | 4.48958 | -2.75003 |
| C | -2.06926 | 5.4232 | -1.74863 |
| C | -1.5207 | 5.08233 | -0.48447 |
| C | -1.06354 | 3.73803 | -0.22415 |
| C | -1.04869 | 2.81841 | -1.33748 |
| C | -1.429 | 6.08056 | 0.52102 |
| C | -0.93075 | 5.78174 | 1.76371 |
| C | -0.5784 | 4.45092 | 2.06331 |
| C | -0.66338 | 3.42791 | 1.12893 |
| C | -0.34812 | 1.49892 | -1.35114 |
| C | -0.44541 | 2.04753 | 1.65552 |
| C | 0.70645 | 1.74549 | 2.39668 |
| C | 0.86096 | 0.48022 | 2.96012 |
| C | -0.12049 | -0.49001 | 2.79172 |
| C | -1.28219 | -0.19632 | 2.05954 |
| C | -1.44707 | 1.07904 | 1.50574 |
| C | -1.01628 | 0.33415 | -1.7578 |
| C | -0.31473 | -0.86424 | -1.86705 |
| C | 1.04831 | -0.94409 | -1.58394 |
| C | 1.7228 | 0.2169 | -1.17843 |
| C | 1.02008 | 1.42576 | -1.07571 |
| O | 3.70943 | -1.96059 | -1.04623 |
| C | 10.17418 | -3.30011 | 0.24741 |
| C | 7.63939 | -0.4566 | -0.04735 |
| C | 8.83273 | -1.14929 | 0.12896 |
| C | 8.88558 | -2.5444 | 0.02508 |
| C | 7.69075 | -3.21137 | -0.26954 |
| C | 6.48338 | -2.5387 | -0.45065 |
| C | 6.44922 | -1.14081 | -0.33852 |
| N | 5.28283 | -0.36424 | -0.49866 |
| C | 4.00293 | -0.79282 | -0.82671 |
| N | 3.10087 | 0.25942 | -0.87685 |
| H | -1.59874 | 2.49937 | -3.36821 |
| H | -2.5872 | 4.74556 | -3.71307 |
| H | -2.42021 | 6.44001 | -1.90279 |
| H | -1.76626 | 7.08587 | 0.28334 |
| H | -0.85452 | 6.54651 | 2.53087 |
| H | -0.28836 | 4.19763 | 3.07832 |
| H | 1.48101 | 2.49672 | 2.51693 |
| H | 0.01384 | -1.47967 | 3.22272 |
| H | -2.08409 | 0.36449 | -1.9446 |
| H | -0.83923 | -1.76445 | -2.17509 |
| H | 1.58764 | -1.87614 | -1.66496 |
| H | 1.54751 | 2.32926 | -0.77864 |
| H | 7.63156 | 0.62848 | 0.03719 |
| H | 9.73921 | -0.5911 | 0.34965 |
| H | 7.69731 | -4.2946 | -0.36524 |
| H | 5.57618 | -3.07622 | -0.68449 |
| H | 5.43377 | 0.63 | -0.4128 |
| H | 3.42787 | 1.16804 | -0.58333 |
| H | 11.04569 | -2.69223 | -0.01451 |
| H | 10.2099 | -4.2146 | -0.35257 |
| H | 10.29001 | -3.59751 | 1.29756 |
| H | 1.75592 | 0.24159 | 3.52727 |
| H | -2.34944 | 1.30496 | 0.95809 |
| N | -2.24089 | -1.22377 | 1.94297 |
| C | -3.36916 | -1.25801 | 1.13339 |
| H | -1.99136 | -2.07329 | 2.42755 |
| N | -4.04059 | -2.46801 | 1.22865 |
| C | -5.21963 | -2.86781 | 0.56423 |
| H | -3.66451 | -3.16404 | 1.85537 |
| C | -5.69372 | -4.16216 | 0.8265 |
| C | -5.93461 | -2.05271 | -0.32551 |
| C | -6.85119 | -4.63233 | 0.21493 |
| H | -5.15364 | -4.80542 | 1.51866 |
| C | -7.57564 | -3.83543 | -0.67958 |
| H | -7.1958 | -5.63845 | 0.44044 |
| C | -7.09144 | -2.5457 | -0.92707 |
| H | -5.58415 | -1.05185 | -0.53043 |
| H | -7.63385 | -1.89724 | -1.61101 |
| C | -8.81403 | -4.35644 | -1.36937 |
| H | -9.32473 | -5.10952 | -0.76137 |
| H | -8.57061 | -4.82637 | -2.33086 |
| H | -9.52587 | -3.55113 | -1.57493 |
| O | -3.73464 | -0.32776 | 0.42672 |

**Supplementary Data 4.** Structure of ***p*-1Urea**, S_0_ [DFT/B3LYP/6-31G (d, p), *E*_sp_ = -1111.41232386 hartree].

| atom | x[Å] | y[Å] | z[Å] |
| --- | --- | --- | --- |
| H | -0.42735 | 1.84713 | 1.82129 |
| H | -2.81227 | 1.30192 | 1.63949 |
| C | -0.73808 | 1.00883 | 1.20076 |
| H | -3.5658 | -2.75336 | 0.86683 |
| H | -5.92912 | -3.4946 | 0.80647 |
| C | -2.08938 | 0.70399 | 1.09336 |
| H | 1.70727 | 1.43218 | 1.28209 |
| C | -4.33525 | -2.05241 | 0.55721 |
| C | -5.68357 | -2.47242 | 0.53373 |
| H | 5.94793 | 2.02004 | 1.35623 |
| H | 3.78285 | 1.48259 | 1.13683 |
| N | 1.57524 | 0.62444 | 0.69146 |
| C | 0.2292 | 0.24253 | 0.53016 |
| C | -2.52958 | -0.37782 | 0.31316 |
| C | 6.21247 | 1.18211 | 0.71394 |
| N | 3.87172 | 0.67806 | 0.53397 |
| C | -3.96732 | -0.75671 | 0.23161 |
| C | -6.67673 | -1.58765 | 0.18795 |
| H | 8.30983 | 1.59526 | 0.83802 |
| C | 7.55014 | 0.9404 | 0.41865 |
| H | -7.7185 | -1.89718 | 0.18073 |
| C | 2.70866 | 0.02617 | 0.1535 |
| C | -0.19118 | -0.84383 | -0.25174 |
| C | 5.20324 | 0.36151 | 0.18812 |
| C | -1.55004 | -1.13585 | -0.34599 |
| C | -4.98625 | 0.17585 | -0.16935 |
| C | -6.35737 | -0.2526 | -0.17457 |
| H | 0.54232 | -1.439 | -0.7754 |
| O | 2.68054 | -0.95913 | -0.57105 |
| C | 7.9312 | -0.12461 | -0.40625 |
| H | -1.86082 | -1.97308 | -0.96413 |
| C | 5.56486 | -0.70797 | -0.64394 |
| C | -7.37057 | 0.66648 | -0.56012 |
| H | 9.81267 | -1.11073 | 0.02454 |
| C | -4.70023 | 1.5016 | -0.60056 |
| H | -8.40441 | 0.3308 | -0.55073 |
| C | 9.38499 | -0.40764 | -0.70158 |
| H | 9.98726 | 0.50518 | -0.66158 |
| C | 6.91171 | -0.93087 | -0.92569 |
| H | 4.797 | -1.34373 | -1.05959 |
| C | -7.05763 | 1.94857 | -0.94695 |
| C | -5.706 | 2.36407 | -0.97656 |
| H | 7.17199 | -1.76292 | -1.57556 |
| H | 9.51228 | -0.8513 | -1.69375 |
| H | -7.84276 | 2.63874 | -1.24153 |
| H | -5.45932 | 3.36907 | -1.3066 |
| H | -3.66783 | 1.82815 | -0.64639 |

**Supplementary Data 5.** Structure of ***p*-2Urea** with two equivalents of AcO^–^, S_0_ [DFT/CAM-B3LYP/6-31G+ (d), *E*_sp_ = -2292.88700236 hartree].

| atom | x[Å] | y[Å] | z[Å] |
| --- | --- | --- | --- |
| C | 8.69305 | -5.54101 | 2.25067 |
| H | 8.33751 | -4.7094 | -0.33413 |
| C | 7.73418 | -4.50038 | 1.72593 |
| C | 7.67696 | -4.19334 | 0.36118 |
| C | 6.86911 | -3.81051 | 2.57053 |
| H | 6.88426 | -4.0229 | 3.63858 |
| C | 6.79907 | -3.24498 | -0.13774 |
| H | 6.77215 | -3.0179 | -1.20373 |
| C | 5.97474 | -2.85039 | 2.09709 |
| C | 5.92987 | -2.55852 | 0.72964 |
| H | 5.31383 | -2.32543 | 2.77276 |
| H | 5.13547 | -1.51748 | -0.88512 |
| N | 5.07308 | -1.62577 | 0.14428 |
| C | 4.19029 | -0.78386 | 0.77269 |
| O | 4.02889 | -0.71635 | 1.99098 |
| H | 3.75832 | -0.15214 | -1.12058 |
| N | 3.49904 | -0.00883 | -0.14133 |
| H | 2.50019 | 0.85855 | 2.2507 |
| C | 2.66453 | 1.07658 | 0.12002 |
| C | 2.22609 | 1.45902 | 1.39506 |
| H | -4.94359 | -2.26836 | 0.98171 |
| H | -6.15989 | -4.10475 | 1.41088 |
| H | -3.92 | -0.6184 | 1.13917 |
| C | 2.25125 | 1.84347 | -0.98096 |
| H | 2.56478 | 1.54543 | -1.97809 |
| C | 1.46897 | 2.61509 | 1.54921 |
| H | -3.1094 | 1.34486 | 1.87421 |
| C | -6.16195 | -4.37052 | 0.35686 |
| H | 1.15521 | 2.89894 | 2.55084 |
| H | -7.3241 | -6.13718 | 0.65986 |
| C | -2.76976 | 1.60109 | 0.87152 |
| N | -3.66598 | -0.48669 | 0.14625 |
| N | -4.85821 | -2.41351 | -0.02993 |
| C | -6.81483 | -5.5142 | -0.07389 |
| H | -2.02982 | 3.51665 | 1.44827 |
| C | -2.16551 | 2.822 | 0.62371 |
| C | 1.4878 | 2.9853 | -0.80833 |
| C | -3.0026 | 0.69964 | -0.17949 |
| C | -5.48617 | -3.53746 | -0.55234 |
| C | 1.10408 | 3.41842 | 0.46645 |
| C | -4.1534 | -1.43789 | -0.71247 |
| C | -1.78459 | 3.20251 | -0.66988 |
| C | -2.57503 | 1.03956 | -1.46909 |
| C | -6.82839 | -5.88407 | -1.42248 |
| H | 1.21998 | 3.57757 | -1.67887 |
| O | -3.9975 | -1.43657 | -1.93356 |
| C | -5.49014 | -3.898 | -1.90601 |
| C | -1.98778 | 2.27993 | -1.697 |
| C | 0.5611 | 4.79164 | 0.65295 |
| C | -1.4177 | 4.61816 | -0.94614 |
| C | -7.55533 | -7.12245 | -1.88596 |
| H | -2.72376 | 0.34102 | -2.28036 |
| C | -6.1531 | -5.0535 | -2.31414 |
| H | 2.02768 | 5.16733 | 2.1443 |
| C | -0.46974 | 5.37432 | -0.16837 |
| H | -2.91059 | 4.71055 | -2.45452 |
| C | -2.18722 | 5.28341 | -1.88249 |
| C | 1.24082 | 5.60663 | 1.53912 |
| H | -4.97119 | -3.27172 | -2.61795 |
| H | -1.68026 | 2.53276 | -2.70927 |
| C | -2.14702 | 6.68359 | -2.03376 |
| C | -0.55722 | 6.8021 | -0.21541 |
| C | 1.02785 | 6.99794 | 1.5998 |
| H | -6.13542 | -5.31223 | -3.37159 |
| C | -1.38983 | 7.43296 | -1.17546 |
| H | -2.77642 | 7.16229 | -2.77972 |
| C | 0.18773 | 7.5916 | 0.6982 |
| H | 1.59014 | 7.59706 | 2.31148 |
| H | -1.41131 | 8.5198 | -1.21145 |
| H | 0.0745 | 8.67279 | 0.66451 |
| H | 8.48931 | -6.5324 | 1.82599 |
| H | 8.62149 | -5.62886 | 3.34034 |
| H | 9.73429 | -5.29466 | 2.00632 |
| H | -7.29262 | -7.99732 | -1.27898 |
| H | -7.30943 | -7.35676 | -2.92719 |
| H | -8.64519 | -7.00301 | -1.82546 |
| C | 5.50284 | -1.34043 | -3.45705 |
| O | 4.79753 | -0.86009 | -2.51249 |
| C | 5.17347 | -0.80704 | -4.85555 |
| O | 6.41136 | -2.18572 | -3.33797 |
| O | -4.38891 | 0.46065 | 3.66132 |
| H | 5.26703 | 0.28444 | -4.86662 |
| H | 4.1323 | -1.04355 | -5.10108 |
| H | 5.83574 | -1.24274 | -5.60803 |
| C | -5.52053 | -1.30292 | 4.81944 |
| C | -4.82826 | -0.70122 | 3.59045 |
| H | -5.56378 | -0.57998 | 5.63791 |
| H | -4.97444 | -2.19492 | 5.1461 |
| H | -6.53443 | -1.62293 | 4.554 |
| O | -4.75639 | -1.47003 | 2.57684 |

**Supplementary Data 6.** Structure of ***m*-2Urea** with two equivalents of AcO^–^, S_0_ [DFT/CAM-B3LYP/6-31G+ (d), *E*_sp_ = -2292.89572959 hartree].

| atom | x[Å] | y[Å] | z[Å] |
| --- | --- | --- | --- |
| C | 1.9452 | 4.48262 | -1.11213 |
| C | 1.8771 | 5.88601 | -1.0555 |
| C | 0.75293 | 6.47609 | -0.55436 |
| C | -0.3107 | 5.68874 | -0.04529 |
| C | -0.2243 | 4.25405 | -0.02261 |
| C | 0.93821 | 3.65958 | -0.6425 |
| C | -1.46224 | 6.35796 | 0.44029 |
| C | -2.51076 | 5.65191 | 0.95587 |
| C | -2.41143 | 4.25297 | 1.05424 |
| C | -1.30891 | 3.54331 | 0.61513 |
| C | 1.15158 | 2.20621 | -0.91568 |
| C | -1.34637 | 2.08432 | 0.93822 |
| C | -0.33809 | 1.47845 | 1.68644 |
| C | -0.47836 | 0.14742 | 2.06858 |
| C | -1.61483 | -0.57457 | 1.73613 |
| C | -2.64637 | 0.02837 | 1.00094 |
| C | -2.49303 | 1.35871 | 0.59681 |
| C | 0.22915 | 1.44914 | -1.64215 |
| C | 0.53908 | 0.13695 | -1.97719 |
| C | 1.75414 | -0.4454 | -1.62647 |
| C | 2.69331 | 0.30979 | -0.9138 |
| C | 2.36882 | 1.62727 | -0.56115 |
| O | 4.00949 | -2.25209 | -1.46998 |
| C | 9.84178 | -5.49594 | -0.69922 |
| C | 8.05137 | -2.20805 | 0.03983 |
| C | 9.02257 | -3.19315 | -0.03266 |
| C | 8.77213 | -4.43356 | -0.63144 |
| C | 7.49983 | -4.64171 | -1.15629 |
| C | 6.50204 | -3.66867 | -1.0982 |
| C | 6.77007 | -2.4354 | -0.49458 |
| N | 5.85092 | -1.39247 | -0.37482 |
| C | 4.56101 | -1.34718 | -0.84388 |
| N | 3.95394 | -0.1479 | -0.52424 |
| H | 2.8059 | 4.01794 | -1.58143 |
| H | 2.69943 | 6.48275 | -1.44066 |
| H | 0.64871 | 7.55843 | -0.53483 |
| H | -1.488 | 7.44398 | 0.38894 |
| H | -3.40112 | 6.15695 | 1.3205 |
| H | -3.21586 | 3.70106 | 1.52862 |
| H | 0.54999 | 2.03648 | 1.96396 |
| H | -1.718 | -1.61204 | 2.04297 |
| H | -0.72209 | 1.88029 | -1.93607 |
| H | -0.18594 | -0.45642 | -2.52785 |
| H | 1.98648 | -1.4658 | -1.89585 |
| H | 3.0941 | 2.20593 | 0.00565 |
| H | 8.26708 | -1.24775 | 0.5086 |
| H | 10.00527 | -2.98889 | 0.38998 |
| H | 7.26753 | -5.59466 | -1.62943 |
| H | 5.52119 | -3.85267 | -1.51411 |
| H | 6.18051 | -0.53505 | 0.10491 |
| H | 4.54056 | 0.5202 | -0.01853 |
| H | 10.72803 | -5.14658 | -1.24428 |
| H | 9.47395 | -6.39346 | -1.20839 |
| H | 10.17823 | -5.79844 | 0.30071 |
| H | 0.31247 | -0.33522 | 2.63595 |
| H | -3.27928 | 1.81796 | 0.01464 |
| N | -3.77335 | -0.75081 | 0.72791 |
| C | -4.94896 | -0.36182 | 0.11557 |
| H | -3.76857 | -1.7022 | 1.10428 |
| N | -5.87784 | -1.37179 | 0.12635 |
| C | -7.16754 | -1.35379 | -0.40675 |
| H | -5.58388 | -2.2413 | 0.6097 |
| C | -7.93872 | -2.51625 | -0.2248 |
| C | -7.72967 | -0.27563 | -1.09894 |
| C | -9.22788 | -2.58409 | -0.727 |
| H | -7.50702 | -3.35814 | 0.31709 |
| C | -9.80612 | -1.51616 | -1.42362 |
| H | -9.80071 | -3.49709 | -0.571 |
| C | -9.03045 | -0.37309 | -1.59358 |
| H | -7.14693 | 0.62363 | -1.24187 |
| H | -9.44618 | 0.47854 | -2.13029 |
| C | -11.21158 | -1.60914 | -1.9653 |
| H | -11.94359 | -1.79038 | -1.16788 |
| H | -11.31514 | -2.42821 | -2.68844 |
| H | -11.50014 | -0.68249 | -2.47352 |
| O | -5.13282 | 0.75345 | -0.3752 |
| C | 7.2053 | 1.55451 | 1.2844 |
| O | 8.27582 | 0.92122 | 1.36356 |
| O | 6.12185 | 1.13199 | 0.76622 |
| C | 7.14825 | 2.97677 | 1.8524 |
| H | 6.86613 | 3.67892 | 1.06013 |
| H | 8.11206 | 3.26862 | 2.27717 |
| H | 6.37242 | 3.03404 | 2.62366 |
| H | -3.8571 | -4.99031 | 3.52869 |
| C | -4.17706 | -5.45104 | 2.58764 |
| H | -4.70621 | -6.38438 | 2.7957 |
| H | -3.2714 | -5.66113 | 2.0085 |
| C | -5.06768 | -4.47456 | 1.81337 |
| O | -4.52958 | -3.35659 | 1.52747 |
| O | -6.22793 | -4.82474 | 1.5208 |

**Supplementary Data 7.** Structure of ***p*-1Urea** with one equivalent of AcO^–^, S_0_ [DFT/CAM-B3LYP/6-31G+ (d), *E*_sp_ = -1339.32176345 hartree].

| atom | x[Å] | y[Å] | z[Å] |
| --- | --- | --- | --- |
| H | 0.65889 | 1.9352 | -0.62529 |
| H | 3.08651 | 1.54084 | -0.7598 |
| C | 1.05782 | 0.93254 | -0.50058 |
| H | 4.12473 | -2.25694 | -2.11469 |
| H | 6.53226 | -2.76271 | -2.41234 |
| C | 2.42108 | 0.70269 | -0.56803 |
| H | -1.39357 | 1.2153 | -0.33923 |
| C | 4.85521 | -1.76322 | -1.48066 |
| C | 6.22764 | -2.04358 | -1.65663 |
| H | -5.62526 | 1.37672 | -0.0386 |
| H | -3.45894 | 1.02711 | -0.15634 |
| N | -1.19152 | 0.2024 | -0.2158 |
| C | 0.1586 | -0.12712 | -0.27518 |
| C | 2.9546 | -0.58529 | -0.42182 |
| C | -5.8167 | 0.31891 | 0.11802 |
| N | -3.45699 | -0.00243 | -0.0182 |
| C | 4.41128 | -0.85191 | -0.54533 |
| C | 7.16773 | -1.3998 | -0.89714 |
| H | -7.93372 | 0.56227 | 0.24575 |
| C | -7.11032 | -0.14952 | 0.27888 |
| H | 8.22794 | -1.5977 | -1.03542 |
| C | -2.251 | -0.65795 | -0.02126 |
| C | 0.67982 | -1.42107 | -0.12848 |
| C | -4.724 | -0.5662 | 0.15397 |
| C | 2.05291 | -1.62988 | -0.20779 |
| C | 5.38111 | -0.19445 | 0.28525 |
| C | 6.76768 | -0.46518 | 0.09171 |
| H | 0.00344 | -2.24496 | 0.04793 |
| O | -2.12553 | -1.87495 | 0.13212 |
| C | -7.38028 | -1.5074 | 0.48158 |
| H | 2.43447 | -2.64063 | -0.08214 |
| C | -4.97824 | -1.92742 | 0.35605 |
| C | 7.72444 | 0.19475 | 0.90772 |
| H | -9.41461 | -1.7775 | -0.22095 |
| C | 5.01321 | 0.70021 | 1.32714 |
| H | 8.77879 | -0.01435 | 0.74169 |
| C | -8.79439 | -2.00373 | 0.65539 |
| H | -9.28272 | -1.54276 | 1.52307 |
| C | -6.29037 | -2.37239 | 0.51514 |
| H | -4.15048 | -2.62129 | 0.3864 |
| C | 7.33566 | 1.06888 | 1.88842 |
| C | 5.9606 | 1.31499 | 2.10555 |
| H | -6.46032 | -3.43648 | 0.67092 |
| H | -8.81587 | -3.08865 | 0.80303 |
| H | 8.07914 | 1.56498 | 2.50638 |
| H | 5.65192 | 1.99106 | 2.89781 |
| H | 3.96094 | 0.88495 | 1.50881 |
| O | -1.48041 | 2.95965 | -0.57255 |
| C | -2.64847 | 3.43553 | -0.54725 |
| C | -2.77717 | 4.94478 | -0.74752 |
| H | -2.18794 | 5.46666 | 0.01383 |
| H | -3.82041 | 5.26349 | -0.69037 |
| H | -2.36094 | 5.2194 | -1.72268 |
| O | -3.70475 | 2.77194 | -0.37368 |

**Supplementary Data 8.** Structure of the ***p*-2Urea** dimer (i) without any AcO^–^ ions, S_0_ [DFT/CAM-B3LYP-GD3/3-21G, *E*_sp_ = -4123.05658780 hartree].

| atom | x[Å] | y[Å] | z[Å] |
| --- | --- | --- | --- |
| C | 0.16611083 | 1.60258071 | -2.0377531 |
| C | 1.30402371 | 1.03652522 | -1.3422027 |
| C | 1.32536808 | 1.16229136 | 0.09731522 |
| C | -0.7928875 | 2.30221601 | -1.3220294 |
| C | -0.7263766 | 2.46253912 | 0.07602699 |
| H | -1.6498122 | 2.6909802 | -1.859053 |
| C | 0.30473359 | 1.88641639 | 0.76998856 |
| H | -1.5153601 | 2.9923809 | 0.59487753 |
| H | 0.35468083 | 1.96378805 | 1.84885633 |
| C | 2.41865692 | 0.35096099 | -1.9557699 |
| C | 2.35833614 | 0.55178547 | 0.8584823 |
| C | 3.36651482 | -0.1469649 | 0.24292141 |
| H | 2.31731096 | 0.63163836 | 1.93910195 |
| C | 3.39678927 | -0.2285778 | -1.1639906 |
| H | 4.22332165 | -0.7290464 | -1.6557073 |
| H | 4.14710216 | -0.6194983 | 0.82758939 |
| C | -0.127691 | 1.42802839 | -3.4947455 |
| C | -0.3187584 | 2.54707552 | -4.3199793 |
| C | -0.2677026 | 0.15490575 | -4.0621349 |
| C | -0.5959309 | 2.40208014 | -5.6781756 |
| H | -0.2079665 | 3.53819532 | -3.8954204 |
| C | 2.5796331 | 0.26095172 | -3.4305725 |
| C | -0.6833329 | 1.12345889 | -6.2368672 |
| H | -0.6996198 | 3.27065971 | -6.3145854 |
| C | -0.5186952 | -7.884E-05 | -5.4189877 |
| H | -0.0989102 | -0.7162554 | -3.4475207 |
| C | 2.59808786 | -0.9807849 | -4.0777655 |
| H | -0.4811105 | -0.9794273 | -5.8800687 |
| C | 2.62254554 | 1.42342775 | -4.2105501 |
| C | 2.55415788 | 1.34756409 | -5.5907441 |
| H | 2.62594778 | 2.38872951 | -3.7204741 |
| C | 2.4384038 | 0.1031857 | -6.2297111 |
| H | 2.47091001 | 2.25102656 | -6.1831228 |
| C | 2.52677074 | -1.0694374 | -5.4664179 |
| H | 2.5779065 | -1.8856559 | -3.4798999 |
| H | 2.42902794 | -2.0284553 | -5.9514613 |
| N | -0.9618789 | 0.92761263 | -7.6181153 |
| N | 2.20025328 | 0.11432533 | -7.6182094 |
| C | -0.3579945 | 1.63859737 | -8.646102 |
| H | -1.440689 | 0.06296825 | -7.8576773 |
| C | 1.47433858 | -0.8668906 | -8.2940566 |
| H | 2.08603472 | 1.05770275 | -8.0092653 |
| O | 0.67010102 | 2.34299332 | -8.4958397 |
| O | 0.85031871 | -1.8045558 | -7.7578019 |
| N | -1.0097632 | 1.45487862 | -9.8487173 |
| N | 1.50603481 | -0.6323992 | -9.6629201 |
| C | -0.5251782 | 1.73721645 | -11.140877 |
| H | -1.9048928 | 0.97499322 | -9.8201279 |
| C | 0.70882505 | -1.2363967 | -10.652863 |
| H | 2.17296369 | 0.05832779 | -9.9955148 |
| C | 0.72040497 | 2.3350219 | -11.390677 |
| C | -0.4400693 | -1.9924818 | -10.367054 |
| C | -1.316423 | 1.35884231 | -12.230464 |
| C | 1.16369618 | 2.48221747 | -12.701777 |
| H | 1.30766618 | 2.67733088 | -10.553787 |
| C | -1.2437256 | -2.4496426 | -11.408256 |
| H | -0.6608807 | -2.2332046 | -9.338703 |
| C | 1.04423946 | -1.0140279 | -11.9933 |
| C | 0.39836517 | 2.06610063 | -13.804173 |
| H | 2.1360129 | 2.93339084 | -12.864644 |
| C | -0.8632914 | 1.52260422 | -13.534228 |
| H | -2.2730453 | 0.87741735 | -12.058581 |
| C | 0.23890351 | -1.489691 | -13.020793 |
| H | 1.91377675 | -0.4154203 | -12.237033 |
| C | -0.9379635 | -2.1994206 | -12.756858 |
| H | -2.1318864 | -3.0186778 | -11.156818 |
| C | 0.96976494 | 2.17841191 | -15.227475 |
| C | -0.0306991 | 1.6857409 | -16.298263 |
| H | -0.2869421 | 0.63169877 | -16.143742 |
| H | 0.42535237 | 1.78290812 | -17.289598 |
| H | -0.9501968 | 2.28148335 | -16.282577 |
| C | 1.32613525 | 3.65608675 | -15.535616 |
| H | 0.42879529 | 4.28066057 | -15.47161 |
| H | 1.7432465 | 3.73687979 | -16.54647 |
| H | 2.06626167 | 4.03908683 | -14.826028 |
| C | 2.24755371 | 1.3013447 | -15.319571 |
| H | 2.00004011 | 0.25604693 | -15.103565 |
| H | 3.00367828 | 1.63399789 | -14.601221 |
| H | 2.67474192 | 1.36169634 | -16.327581 |
| C | -1.9018837 | -2.6495016 | -13.867304 |
| C | -3.2429941 | -1.8868424 | -13.693894 |
| H | -3.6867687 | -2.0984277 | -12.715538 |
| H | -3.9541288 | -2.1877475 | -14.472553 |
| H | -3.0695668 | -0.8075109 | -13.771412 |
| C | -2.1521823 | -4.1766707 | -13.76708 |
| H | -2.5874416 | -4.4429761 | -12.79886 |
| H | -1.2087678 | -4.7203323 | -13.88397 |
| H | -2.8451902 | -4.4945584 | -14.555222 |
| C | -1.3507102 | -2.3413447 | -15.27891 |
| H | -1.2001082 | -1.2652147 | -15.419043 |
| H | -2.0702874 | -2.6848777 | -16.030262 |
| H | -0.3993583 | -2.8571753 | -15.450056 |
| H | -1.4996166 | 1.18525583 | -14.340765 |
| H | 0.5274972 | -1.2637236 | -14.037081 |
| H | -2.47091 | -2.2510266 | 6.18312278 |
| H | -3.0036783 | -1.6339979 | 14.6012212 |
| H | -1.9137768 | 0.41542031 | 12.2370327 |
| H | -2.6259478 | -2.3887295 | 3.72047411 |
| H | -2.0860347 | -1.0577028 | 8.00926533 |
| H | -2.6747419 | -1.3616963 | 16.327581 |
| C | -2.5541579 | -1.3475641 | 5.59074414 |
| C | -2.2475537 | -1.3013447 | 15.3195712 |
| H | -2.1729637 | -0.0583278 | 9.99551484 |
| H | -2.0000401 | -0.2560469 | 15.103565 |
| C | -2.6225455 | -1.4234278 | 4.21055005 |
| C | -1.0442395 | 1.01402786 | 11.9933002 |
| H | -0.5274972 | 1.26372363 | 14.0370809 |
| N | -2.2002533 | -0.1143253 | 7.61820941 |
| H | 0.39935828 | 2.85717528 | 15.4500559 |
| N | -1.5060348 | 0.63239915 | 9.66292008 |
| C | -0.2389035 | 1.48969095 | 13.0207933 |
| C | -2.4384038 | -0.1031857 | 6.22971107 |
| H | -2.1360129 | -2.9333908 | 12.8646439 |
| C | -0.7088251 | 1.23639672 | 10.6528631 |
| C | -1.4743386 | 0.86689057 | 8.2940566 |
| H | 1.20876779 | 4.7203323 | 13.8839704 |
| H | 0.20796649 | -3.5381953 | 3.89542043 |
| C | -2.5796331 | -0.2609517 | 3.43057246 |
| C | 1.35071017 | 2.34134472 | 15.2789096 |
| C | -1.1636962 | -2.4822175 | 12.7017769 |
| C | -3.3967893 | 0.22857783 | 1.16399059 |
| H | -4.2233217 | 0.72904637 | 1.65570732 |
| H | -1.3076662 | -2.6773309 | 10.5537868 |
| H | -2.0662617 | -4.0390868 | 14.8260277 |
| C | -3.3665148 | 0.14696494 | -0.2429214 |
| C | -2.4186569 | -0.350961 | 1.95576986 |
| C | -0.9697649 | -2.1784119 | 15.227475 |
| H | -4.1471022 | 0.61949825 | -0.8275894 |
| H | 0.69961975 | -3.2706597 | 6.31458543 |
| C | -2.3583361 | -0.5517855 | -0.8584823 |
| C | -0.720405 | -2.3350219 | 11.3906769 |
| C | 0.31875841 | -2.5470755 | 4.31997932 |
| O | -0.670101 | -2.3429933 | 8.49583965 |
| C | 0.93796347 | 2.19942061 | 12.7568581 |
| H | -1.7432465 | -3.7368798 | 16.5464697 |
| H | 1.20010816 | 1.26521467 | 15.4190433 |
| C | -2.5267707 | 1.06943736 | 5.46641792 |
| C | -1.3261353 | -3.6560868 | 15.5356163 |
| C | -1.3253681 | -1.1622914 | -0.0973152 |
| C | -1.3040237 | -1.0365252 | 1.34220273 |
| C | 2.15218229 | 4.17667073 | 13.7670797 |
| H | 2.07028743 | 2.68487766 | 16.0302617 |
| C | 0.59593093 | -2.4020801 | 5.67817559 |
| H | -2.317311 | -0.6316384 | -1.939102 |
| C | -0.3983652 | -2.0661006 | 13.804173 |
| C | -2.5980879 | 0.9807849 | 4.07776554 |
| C | 0.44006932 | 1.9924818 | 10.3670535 |
| C | -0.3047336 | -1.8864164 | -0.7699886 |
| O | -0.8503187 | 1.80455581 | 7.75780186 |
| C | 1.90188372 | 2.64950162 | 13.8673037 |
| C | -0.1661108 | -1.6025807 | 2.03775312 |
| C | 0.7263766 | -2.4625391 | -0.076027 |
| H | -0.3546808 | -1.9637881 | -1.8488563 |
| H | -0.4253524 | -1.7829081 | 17.2895978 |
| C | 0.79288746 | -2.302216 | 1.32202943 |
| C | 1.24372557 | 2.44964259 | 11.4082557 |
| C | 0.03069905 | -1.6857409 | 16.2982627 |
| C | 0.12769097 | -1.4280284 | 3.49474553 |
| H | 0.28694209 | -0.6316988 | 16.1437416 |
| H | 2.84519019 | 4.49455837 | 14.5552225 |
| H | -2.4290279 | 2.02845526 | 5.95146127 |
| C | 0.35799449 | -1.6385974 | 8.64610195 |
| H | 1.51536014 | -2.9923809 | -0.5948775 |
| C | 0.52517823 | -1.7372165 | 11.1408773 |
| H | 2.58744156 | 4.44297609 | 12.7988598 |
| H | 0.6608807 | 2.23320455 | 9.338703 |
| H | 1.64981218 | -2.6909802 | 1.85905301 |
| C | 0.6833329 | -1.1234589 | 6.23686718 |
| C | 0.86329137 | -1.5226042 | 13.534228 |
| H | -2.5779065 | 1.88565593 | 3.47989985 |
| H | -0.4287953 | -4.2806606 | 15.4716101 |
| N | 1.00976318 | -1.4548786 | 9.84871732 |
| N | 0.96187888 | -0.9276126 | 7.61811532 |
| C | 1.31642301 | -1.3588423 | 12.230464 |
| C | 0.26770257 | -0.1549058 | 4.06213486 |
| H | 2.13188637 | 3.01867775 | 11.1568183 |
| C | 3.24299407 | 1.88684243 | 13.6938942 |
| C | 0.51869518 | 0.00007884 | 5.41898772 |
| H | 0.9501968 | -2.2814834 | 16.2825768 |
| H | 1.49961662 | -1.1852558 | 14.3407653 |
| H | 3.06956681 | 0.80751088 | 13.7714116 |
| H | 1.90489279 | -0.9749932 | 9.82012794 |
| H | 3.9541288 | 2.18774747 | 14.472553 |
| H | 1.440689 | -0.0629683 | 7.85767733 |
| H | 0.09891017 | 0.71625539 | 3.44752066 |
| H | 2.27304527 | -0.8774174 | 12.058581 |
| H | 3.68676873 | 2.09842773 | 12.7155376 |
| H | 0.48111053 | 0.9794273 | 5.88006871 |

**Supplementary Data 9.** Structure of the ***p*-2Urea** dimer (ii) with two AcO^–^ ions, S_0_ [DFT/CAM-B3LYP-GD3/3-21G, *E*_sp_ = -4123.10730799 hartree].

| atom | x[Å] | y[Å] | z[Å] |
| --- | --- | --- | --- |
| C | -1.1294598 | -0.2780584 | 8.24973684 |
| C | -0.3806345 | 0.30519334 | 9.34191992 |
| C | -0.9611616 | 0.20371281 | 10.6633887 |
| C | -2.3713145 | -0.8455233 | 8.49062302 |
| C | -2.9372965 | -0.9015061 | 9.78076615 |
| H | -2.9031763 | -1.2848703 | 7.65442624 |
| C | -2.2366441 | -0.3971896 | 10.84698 |
| H | -3.9088842 | -1.359814 | 9.92262189 |
| H | -2.6410238 | -0.4498349 | 11.852176 |
| C | 0.89614345 | 0.97755859 | 9.23490568 |
| C | -0.2616021 | 0.70045584 | 11.7973721 |
| C | 0.97051644 | 1.28871876 | 11.6622215 |
| H | -0.7263834 | 0.60233186 | 12.7725756 |
| C | 1.53537325 | 1.43002048 | 10.3777638 |
| H | 2.48937346 | 1.93170143 | 10.2645772 |
| H | 1.50315162 | 1.66339677 | 12.5283059 |
| C | -0.6541354 | -0.3486564 | 6.83453543 |
| C | -1.3919669 | 0.28422627 | 5.82181154 |
| C | 0.49868525 | -1.0600857 | 6.48496117 |
| C | -0.9522153 | 0.25547453 | 4.50226426 |
| H | -2.2880715 | 0.83545998 | 6.08323947 |
| C | 1.58643271 | 1.22303585 | 7.93692527 |
| C | 0.23592787 | -0.4012918 | 4.17556856 |
| H | -1.5036503 | 0.76627962 | 3.72676278 |
| C | 0.95853955 | -1.0621739 | 5.1708448 |
| H | 1.07327817 | -1.5524236 | 7.25736363 |
| C | 2.82574099 | 0.62451028 | 7.67691876 |
| H | 1.92198355 | -1.4870557 | 4.92401971 |
| C | 0.99365797 | 2.00349215 | 6.93617936 |
| C | 1.56107277 | 2.08021227 | 5.67360948 |
| H | 0.04089008 | 2.47960201 | 7.12885067 |
| C | 2.74890668 | 1.38883756 | 5.38983783 |
| H | 1.02743705 | 2.55277237 | 4.85922373 |
| C | 3.4106724 | 0.70320433 | 6.41467607 |
| H | 3.29687077 | 0.0299049 | 8.45150915 |
| H | 4.31651383 | 0.15880299 | 6.19546531 |
| N | 0.71616535 | -0.4200902 | 2.82701595 |
| N | 3.19189707 | 1.36219182 | 4.04290079 |
| C | 0.83399641 | 0.72727976 | 2.08080517 |
| H | 0.74066312 | -1.3266286 | 2.36272495 |
| C | 3.71431942 | 0.21869154 | 3.44621127 |
| H | 2.66590735 | 1.98559817 | 3.42573 |
| O | 0.664656 | 1.88274557 | 2.55385211 |
| O | 3.86333306 | -0.8821416 | 4.00593447 |
| N | 1.18850996 | 0.50006829 | 0.76507952 |
| N | 4.02955026 | 0.44151033 | 2.10400662 |
| C | 1.19584653 | 1.464524 | -0.2624392 |
| H | 1.51285844 | -0.4320007 | 0.51319021 |
| C | 4.0606767 | -0.5452956 | 1.10123009 |
| H | 4.14663881 | 1.40301681 | 1.79682824 |
| C | 0.41707934 | 2.63338424 | -0.221858 |
| C | 3.42985046 | -1.7970109 | 1.24716363 |
| C | 1.94525764 | 1.20924474 | -1.4173486 |
| C | 0.37872304 | 3.48451124 | -1.3244298 |
| H | -0.1311313 | 2.87322632 | 0.67284683 |
| C | 3.29712566 | -2.6442982 | 0.15133361 |
| H | 3.06475042 | -2.088589 | 2.21974365 |
| C | 4.62140964 | -0.2333571 | -0.1415307 |
| C | 1.12067665 | 3.2347186 | -2.4932529 |
| H | -0.239735 | 4.37270008 | -1.2612446 |
| C | 1.92044812 | 2.0853527 | -2.499138 |
| H | 2.54904883 | 0.31446209 | -1.4671101 |
| C | 4.49155207 | -1.0998175 | -1.2283649 |
| H | 5.12499141 | 0.71772015 | -0.2753656 |
| C | 3.79662337 | -2.3108739 | -1.1222456 |
| H | 2.78617089 | -3.5883558 | 0.29688157 |
| C | 1.05298743 | 4.20825665 | -3.6817955 |
| C | 1.7699443 | 3.64570494 | -4.9328193 |
| H | 2.84031672 | 3.50454286 | -4.7437388 |
| H | 1.66492198 | 4.3614111 | -5.7558275 |
| H | 1.32834559 | 2.69526435 | -5.2454452 |
| C | -0.4275776 | 4.48046113 | -4.0580283 |
| H | -0.9160634 | 3.54731418 | -4.3505849 |
| H | -0.470288 | 5.17614511 | -4.9039239 |
| H | -0.9790861 | 4.92570543 | -3.2237162 |
| C | 1.73525616 | 5.53895182 | -3.267721 |
| H | 2.78452691 | 5.35986433 | -3.0088221 |
| H | 1.23332224 | 5.97765555 | -2.3985559 |
| H | 1.69190245 | 6.25589904 | -4.09635 |
| C | 3.62693212 | -3.2800716 | -2.3054767 |
| C | 2.15050953 | -3.7386572 | -2.4318029 |
| H | 1.82097146 | -4.2601879 | -1.5267904 |
| H | 2.06018599 | -4.4408393 | -3.2690796 |
| H | 1.47026718 | -2.9010422 | -2.6117631 |
| C | 4.5291905 | -4.5179084 | -2.0517057 |
| H | 4.2418119 | -5.0174381 | -1.1204529 |
| H | 5.57785428 | -4.2119293 | -1.9713752 |
| H | 4.42844688 | -5.2318468 | -2.8779099 |
| C | 4.04356572 | -2.6340409 | -3.6482411 |
| H | 3.45516806 | -1.73154 | -3.8460758 |
| H | 3.86262434 | -3.3463381 | -4.4603654 |
| H | 5.10787298 | -2.3747424 | -3.6559602 |
| H | 2.49765215 | 1.83027137 | -3.3754417 |
| H | 4.92417598 | -0.8001024 | -2.1736854 |
| C | 1.12945984 | 0.27805842 | -8.2497368 |
| C | 0.38063454 | -0.3051933 | -9.3419199 |
| C | 0.96116155 | -0.2037128 | -10.663389 |
| C | 2.37131454 | 0.84552333 | -8.490623 |
| C | 2.93729654 | 0.90150612 | -9.7807662 |
| H | 2.90317629 | 1.28487031 | -7.6544262 |
| C | 2.23664405 | 0.39718963 | -10.84698 |
| H | 3.90888423 | 1.35981404 | -9.9226219 |
| H | 2.64102375 | 0.44983488 | -11.852176 |
| C | -0.8961435 | -0.9775586 | -9.2349057 |
| C | 0.26160214 | -0.7004558 | -11.797372 |
| C | -0.9705164 | -1.2887188 | -11.662222 |
| H | 0.72638335 | -0.6023319 | -12.772576 |
| C | -1.5353733 | -1.4300205 | -10.377764 |
| H | -2.4893735 | -1.9317014 | -10.264577 |
| H | -1.5031516 | -1.6633968 | -12.528306 |
| C | 0.6541354 | 0.34865644 | -6.8345354 |
| C | 1.3919669 | -0.2842263 | -5.8218115 |
| C | -0.4986853 | 1.06008574 | -6.4849612 |
| C | 0.95221532 | -0.2554745 | -4.5022643 |
| H | 2.28807147 | -0.83546 | -6.0832395 |
| C | -1.5864327 | -1.2230359 | -7.9369253 |
| C | -0.2359279 | 0.40129178 | -4.1755686 |
| H | 1.50365032 | -0.7662796 | -3.7267628 |
| C | -0.9585396 | 1.06217387 | -5.1708448 |
| H | -1.0732782 | 1.55242362 | -7.2573636 |
| C | -2.825741 | -0.6245103 | -7.6769188 |
| H | -1.9219836 | 1.48705565 | -4.9240197 |
| C | -0.993658 | -2.0034922 | -6.9361794 |
| C | -1.5610728 | -2.0802123 | -5.6736095 |
| H | -0.0408901 | -2.479602 | -7.1288507 |
| C | -2.7489067 | -1.3888376 | -5.3898378 |
| H | -1.0274371 | -2.5527724 | -4.8592237 |
| C | -3.4106724 | -0.7032043 | -6.4146761 |
| H | -3.2968708 | -0.0299049 | -8.4515092 |
| H | -4.3165138 | -0.158803 | -6.1954653 |
| N | -0.7161654 | 0.42009019 | -2.827016 |
| N | -3.1918971 | -1.3621918 | -4.0429008 |
| C | -0.8339964 | -0.7272798 | -2.0808052 |
| H | -0.7406631 | 1.32662862 | -2.362725 |
| C | -3.7143194 | -0.2186915 | -3.4462113 |
| H | -2.6659074 | -1.9855982 | -3.42573 |
| O | -0.664656 | -1.8827456 | -2.5538521 |
| O | -3.8633331 | 0.88214161 | -4.0059345 |
| N | -1.18851 | -0.5000683 | -0.7650795 |
| N | -4.0295503 | -0.4415103 | -2.1040066 |
| C | -1.1958465 | -1.464524 | 0.26243915 |
| H | -1.5128584 | 0.43200068 | -0.5131902 |
| C | -4.0606767 | 0.54529562 | -1.1012301 |
| H | -4.1466388 | -1.4030168 | -1.7968282 |
| C | -0.4170793 | -2.6333842 | 0.22185795 |
| C | -3.4298505 | 1.7970109 | -1.2471636 |
| C | -1.9452576 | -1.2092447 | 1.4173486 |
| C | -0.378723 | -3.4845112 | 1.32442981 |
| H | 0.13113128 | -2.8732263 | -0.6728468 |
| C | -3.2971257 | 2.6442982 | -0.1513336 |
| H | -3.0647504 | 2.08858903 | -2.2197437 |
| C | -4.6214096 | 0.23335706 | 0.14153068 |
| C | -1.1206767 | -3.2347186 | 2.49325293 |
| H | 0.23973501 | -4.3727001 | 1.2612446 |
| C | -1.9204481 | -2.0853527 | 2.49913802 |
| H | -2.5490488 | -0.3144621 | 1.46711013 |
| C | -4.4915521 | 1.09981751 | 1.22836493 |
| H | -5.1249914 | -0.7177202 | 0.27536556 |
| C | -3.7966234 | 2.3108739 | 1.12224556 |
| H | -2.7861709 | 3.58835584 | -0.2968816 |
| C | -1.0529874 | -4.2082567 | 3.68179549 |
| C | -1.7699443 | -3.6457049 | 4.93281934 |
| H | -2.8403167 | -3.5045429 | 4.74373884 |
| H | -1.664922 | -4.3614111 | 5.75582748 |
| H | -1.3283456 | -2.6952644 | 5.24544517 |
| C | 0.42757764 | -4.4804611 | 4.05802826 |
| H | 0.91606338 | -3.5473142 | 4.35058494 |
| H | 0.47028803 | -5.1761451 | 4.90392389 |
| H | 0.97908606 | -4.9257054 | 3.22371623 |
| C | -1.7352562 | -5.5389518 | 3.26772096 |
| H | -2.7845269 | -5.3598643 | 3.00882212 |
| H | -1.2333222 | -5.9776556 | 2.39855587 |
| H | -1.6919025 | -6.255899 | 4.09635001 |
| C | -3.6269321 | 3.28007161 | 2.30547665 |
| C | -2.1505095 | 3.73865717 | 2.43180287 |
| H | -1.8209715 | 4.26018794 | 1.52679037 |
| H | -2.060186 | 4.44083927 | 3.26907958 |
| H | -1.4702672 | 2.90104222 | 2.61176313 |
| C | -4.5291905 | 4.51790842 | 2.05170573 |
| H | -4.2418119 | 5.01743814 | 1.12045292 |
| H | -5.5778543 | 4.21192934 | 1.97137516 |
| H | -4.4284469 | 5.23184675 | 2.87790994 |
| C | -4.0435657 | 2.63404086 | 3.64824107 |
| H | -3.4551681 | 1.73153995 | 3.8460758 |
| H | -3.8626243 | 3.34633814 | 4.46036537 |
| H | -5.107873 | 2.37474239 | 3.65596018 |
| H | -2.4976522 | -1.8302714 | 3.37544165 |
| H | -4.924176 | 0.80010243 | 2.17368542 |

**Supplementary Data 10.** Structure of the ***p*-2Urea** dimer (i) without any AcO^–^ ions, S_0_ [DFT/CAM-B3LYP-GD3/3-21G, *E*_sp_ = -5032.11491557 hartree].

| atom | x[Å] | y[Å] | z[Å] |
| --- | --- | --- | --- |
| C | -2.4878475 | -0.6027748 | 1.72634698 |
| C | -1.8676367 | 0.60923756 | 1.24950336 |
| C | -0.4537771 | 0.77891765 | 1.4869451 |
| C | -1.763401 | -1.4379973 | 2.56993176 |
| C | -0.4207634 | -1.1810376 | 2.91387094 |
| H | -2.2216712 | -2.3672494 | 2.88834036 |
| C | 0.2376536 | -0.1228052 | 2.33862104 |
| H | 0.11636653 | -1.8863561 | 3.53684605 |
| H | 1.30057016 | 0.02284 | 2.49019562 |
| C | -2.5600428 | 1.65088531 | 0.52919738 |
| C | 0.25828817 | 1.82916742 | 0.84654755 |
| C | -0.4053453 | 2.71940882 | 0.03758652 |
| H | 1.33308689 | 1.88567364 | 0.98225855 |
| C | -1.8104347 | 2.65221637 | -0.079154 |
| H | -2.3391418 | 3.44817287 | -0.591078 |
| H | 0.13916178 | 3.5095784 | -0.467884 |
| C | -3.7728324 | -1.1432082 | 1.19760598 |
| C | -4.6859214 | -1.7996637 | 2.03649911 |
| C | -4.0030684 | -1.2065207 | -0.1897006 |
| C | -5.7564057 | -2.5318926 | 1.53100795 |
| H | -4.5500877 | -1.7351517 | 3.11145972 |
| C | -4.037878 | 1.84772268 | 0.57223384 |
| C | -5.9476494 | -2.638645 | 0.13966033 |
| H | -6.4489543 | -3.0443851 | 2.17841651 |
| C | -5.0612737 | -1.9367004 | -0.7107805 |
| H | -3.3248658 | -0.6867956 | -0.8562252 |
| C | -4.75129 | 2.28540855 | -0.5541403 |
| H | -5.2196798 | -2.0055635 | -1.7809743 |
| C | -4.7228804 | 1.8106935 | 1.80061136 |
| C | -6.0362304 | 2.2428627 | 1.90599816 |
| H | -4.1970111 | 1.46316188 | 2.68181922 |
| C | -6.7308429 | 2.73625047 | 0.77729845 |
| H | -6.5499727 | 2.24160153 | 2.86057191 |
| C | -6.0714823 | 2.72010361 | -0.467061 |
| H | -4.2568089 | 2.28783051 | -1.5204083 |
| H | -6.6113508 | 3.0686112 | -1.3322653 |
| N | -6.9429892 | -3.4073977 | -0.4732463 |
| N | -8.0229416 | 3.23024649 | 0.99397525 |
| C | -7.8558095 | -4.2251647 | 0.15838132 |
| H | -6.9269623 | -3.3725382 | -1.5338716 |
| C | -8.83988 | 3.83398994 | 0.06223701 |
| H | -8.3448583 | 3.1728931 | 2.00151159 |
| O | -7.9610668 | -4.3635105 | 1.40397712 |
| O | -8.5649487 | 3.97404668 | -1.1564671 |
| N | -8.6538066 | -4.9028935 | -0.7655312 |
| N | -10.023233 | 4.29247991 | 0.646707 |
| C | -9.6486797 | -5.8206275 | -0.4727745 |
| H | -8.5075093 | -4.7463489 | -1.8093775 |
| C | -11.049756 | 4.96325099 | 0.00321876 |
| H | -10.187185 | 4.15678794 | 1.68958164 |
| C | -10.041829 | -6.2093471 | 0.82889814 |
| C | -11.07981 | 5.27721789 | -1.3753883 |
| C | -10.323324 | -6.4213357 | -1.5628338 |
| C | -11.052471 | -7.1463066 | 1.00825754 |
| H | -9.5286609 | -5.7527693 | 1.66005846 |
| C | -12.161861 | 5.96000627 | -1.9179506 |
| H | -10.238051 | 4.96782279 | -1.9740698 |
| C | -12.15523 | 5.37147104 | 0.78776702 |
| C | -11.726849 | -7.7484063 | -0.0681698 |
| H | -11.322336 | -7.4139002 | 2.02641716 |
| C | -11.331205 | -7.3563992 | -1.3576137 |
| H | -10.025582 | -6.1285556 | -2.5633222 |
| C | -13.226045 | 6.0535657 | 0.22163394 |
| H | -12.137425 | 5.13541824 | 1.84571569 |
| C | -13.263433 | 6.36922022 | -1.1464409 |
| H | -12.14273 | 6.1784956 | -2.9823111 |
| C | -12.836472 | -8.7805994 | 0.19866786 |
| C | -13.455852 | -9.3283366 | -1.1083658 |
| H | -13.904223 | -8.5182959 | -1.6941707 |
| H | -14.238764 | -10.058061 | -0.8643443 |
| H | -12.694478 | -9.8247574 | -1.7202536 |
| C | -12.250927 | -9.9801665 | 0.99126729 |
| H | -11.453801 | -10.454303 | 0.40829483 |
| H | -13.034117 | -10.722653 | 1.20055917 |
| H | -11.825167 | -9.6419594 | 1.94098293 |
| C | -13.971392 | -8.1218264 | 1.02795122 |
| H | -14.393748 | -7.2783334 | 0.47124635 |
| H | -13.583172 | -7.7433117 | 1.97851263 |
| H | -14.76776 | -8.8501456 | 1.2372311 |
| C | -14.430319 | 7.12247255 | -1.8087886 |
| C | -15.05889 | 6.23649543 | -2.9177294 |
| H | -14.310985 | 5.97780356 | -3.673499 |
| H | -15.888155 | 6.76513659 | -3.4089133 |
| H | -15.436141 | 5.30680913 | -2.4782587 |
| C | -13.906829 | 8.44073743 | -2.4398148 |
| H | -13.133034 | 8.22958096 | -3.1843043 |
| H | -13.468778 | 9.07465966 | -1.661084 |
| H | -14.726828 | 8.98579556 | -2.9286179 |
| C | -15.543951 | 7.48557892 | -0.7988476 |
| H | -15.962582 | 6.58203281 | -0.3419609 |
| H | -16.349563 | 8.01964937 | -1.3191831 |
| H | -15.15247 | 8.13184336 | -0.0053004 |
| H | -11.812424 | -7.7829713 | -2.2299984 |
| H | -14.045009 | 6.34047426 | 0.87098351 |
| H | -9.2285392 | 3.45761527 | 6.08857236 |
| C | -10.136617 | 3.37944059 | 5.48565754 |
| H | -10.567972 | 2.38239848 | 5.64257599 |
| O | -8.6573402 | 3.05839609 | 3.61098301 |
| C | -9.7794069 | 3.53676591 | 3.98981708 |
| H | -10.872634 | 4.13392296 | 5.77258493 |
| O | -10.654459 | 4.10310791 | 3.24089329 |
| H | -7.9058169 | -4.6082816 | -5.8918162 |
| H | -7.9138415 | -2.8344101 | -5.6916709 |
| C | -7.4195575 | -3.7674883 | -5.3927077 |
| C | -7.5388175 | -3.924967 | -3.8607212 |
| O | -6.7288459 | -3.2411065 | -3.1472754 |
| O | -8.4612901 | -4.7030151 | -3.4232365 |
| H | -6.3647426 | -3.6955684 | -5.6704604 |
| H | 9.02073401 | -3.7782931 | -5.9141537 |
| H | 10.7916547 | -4.1212667 | -5.8365608 |
| H | 10.1852582 | -2.4447192 | -5.8047102 |
| C | 9.98138766 | -3.4761269 | -5.4896395 |
| C | 9.90058542 | -3.5179111 | -3.9468719 |
| O | 8.78811327 | -3.179527 | -3.4181076 |
| O | 10.9676173 | -3.8547811 | -3.3178176 |
| H | 11.5997271 | 8.30086044 | -3.4644699 |
| H | 9.5767213 | 9.89675809 | -3.2225518 |
| H | 9.45974741 | 7.61448601 | -3.0769782 |
| H | 6.69604033 | -2.6924798 | -2.385369 |
| H | 3.79135406 | 0.75142579 | -2.5562905 |
| H | 12.8263859 | -4.4292833 | -2.1221512 |
| H | 12.6875976 | 9.64224243 | -3.0234963 |
| C | 12.0744573 | 8.82064952 | -2.626663 |
| H | -0.8208606 | -0.1195693 | -2.0907953 |
| H | 10.6931457 | 11.2160688 | -2.7858038 |
| C | 10.0949815 | 10.3828867 | -2.3902676 |
| H | 8.75582547 | -3.1708197 | -1.7787808 |
| H | 1.41642704 | 0.90747827 | -1.9276699 |
| H | 10.7724484 | -3.7977785 | -1.7122402 |
| H | 8.06777413 | 5.73136171 | -2.2860598 |
| H | 4.28599273 | -2.3788142 | -1.8648849 |
| C | -0.035205 | -0.637898 | -1.5532878 |
| H | 5.49840848 | 2.49481028 | -2.0653159 |
| C | 9.41492124 | 7.40411235 | -2.0117559 |
| C | 1.25241166 | -0.068648 | -1.4885998 |
| C | 6.34565289 | -2.6916335 | -1.3595126 |
| H | 15.0597801 | -5.1549781 | -1.3964137 |
| C | 4.08205364 | 0.95021594 | -1.5296747 |
| C | 13.0556004 | -4.5288121 | -1.0672217 |
| O | 6.84296776 | 4.12772987 | -1.6626149 |
| H | 12.7230357 | 8.10803864 | -2.1057512 |
| C | 8.62546356 | 6.33999631 | -1.5923807 |
| C | 5.0016218 | -2.5188403 | -1.0635545 |
| C | 5.04083521 | 1.92641541 | -1.2721873 |
| C | 11.0026541 | 9.36718363 | -1.6457146 |
| H | 9.34048987 | 10.7783154 | -1.7014945 |
| C | 14.3134045 | -4.9392908 | -0.6407358 |
| C | -0.3230439 | -1.759483 | -0.8158216 |
| N | 8.60909272 | -3.1794131 | -0.7291167 |
| N | 10.8047019 | -3.8304064 | -0.6483421 |
| C | 2.28342958 | -0.6412343 | -0.7529479 |
| H | 16.6093359 | -6.6185315 | -0.5648016 |
| H | -1.3370844 | -2.1373333 | -0.7489292 |
| C | 10.1466974 | 8.20319825 | -1.1162355 |
| H | 17.1527216 | -4.9229451 | -0.5336166 |
| C | 7.29210935 | -2.9175632 | -0.3330387 |
| C | 3.47778927 | 0.21370541 | -0.5001199 |
| C | 12.0295527 | -4.2284656 | -0.1391938 |
| C | 16.9925573 | -5.8179045 | 0.07770129 |
| C | 6.96935549 | 4.09767756 | -0.4120233 |
| C | 0.69765725 | -2.4076367 | -0.0710644 |
| C | 2.05116177 | -1.9056384 | -0.0996023 |
| C | 9.66130567 | -3.5227649 | 0.09277511 |
| H | 12.3291028 | 10.9388497 | -0.9397907 |
| C | 11.7340939 | 10.1220287 | -0.5111387 |
| C | 8.52930614 | 6.01804754 | -0.2188681 |
| C | 4.53533636 | -2.5639652 | 0.26296134 |
| C | 5.42770136 | 2.20889296 | 0.05252514 |
| H | 17.9580233 | -6.1345357 | 0.49312867 |
| C | 14.6259098 | -5.0749497 | 0.72186186 |
| C | 3.07619479 | -2.6931177 | 0.54530939 |
| C | 0.37203705 | -3.5304647 | 0.73603529 |
| N | 7.77242535 | 4.98427262 | 0.30739557 |
| N | 6.33532764 | 3.20473322 | 0.42631345 |
| C | 10.0457455 | 7.87691504 | 0.24610476 |
| C | 16.0067001 | -5.5229762 | 1.23230885 |
| C | 6.84237598 | -2.8933318 | 1.00140592 |
| C | 3.90056194 | 0.4712013 | 0.81767005 |
| H | 12.4061069 | 9.44852923 | 0.03221347 |
| H | -0.6636781 | -3.848839 | 0.77711781 |
| C | 12.3362204 | -4.362821 | 1.23482417 |
| H | 11.0154568 | 10.5485981 | 0.19747839 |
| C | 9.26244861 | 6.8178356 | 0.69021337 |
| C | 5.48904559 | -2.7233252 | 1.2802514 |
| O | 9.61080438 | -3.5721693 | 1.34815851 |
| C | 4.8496148 | 1.44434874 | 1.09318955 |
| H | 15.4280742 | -7.6140573 | 1.45641374 |
| C | 2.69357996 | -3.7746651 | 1.33130203 |
| C | 13.601591 | -4.7739492 | 1.63632999 |
| C | 1.34522593 | -4.1698936 | 1.46435562 |
| H | 16.7386194 | -3.4894422 | 1.52377168 |
| C | 15.8553948 | -6.8176435 | 2.07553089 |
| H | 10.5856313 | 8.45426965 | 0.98755023 |
| C | 16.6220359 | -4.4041062 | 2.1149834 |
| H | 7.56948933 | -3.0312999 | 1.78477331 |
| H | 3.46193876 | -0.0993513 | 1.62774602 |
| H | 11.5550976 | -4.1364358 | 1.94281078 |
| H | 7.83316891 | 4.90392571 | 1.36758268 |
| H | 3.47134469 | -4.378025 | 1.78504667 |
| H | 6.49571284 | 3.27454797 | 1.47286916 |
| H | 16.8329183 | -7.1442514 | 2.45782635 |
| H | 1.09512842 | -5.0184027 | 2.09286158 |
| H | 17.6049308 | -4.712696 | 2.49853407 |
| H | 9.19174779 | 6.57671024 | 1.74481844 |
| H | 5.15873863 | -2.7200738 | 2.31423841 |
| H | 5.15748748 | 1.65373168 | 2.11124545 |
| H | 13.7930596 | -4.8604393 | 2.70256745 |
| H | 15.1859707 | -6.647699 | 2.92440687 |
| H | 15.9681157 | -4.1808212 | 2.96353839 |
| O | 8.06978717 | 4.99989494 | 2.96542432 |
| O | 6.59231758 | 3.26391688 | 3.10224258 |
| C | 7.38297061 | 4.12932362 | 3.61123779 |
| C | 7.55551948 | 4.09503419 | 5.14624157 |
| H | 8.24509247 | 3.28185365 | 5.40628339 |
| H | 7.96948533 | 5.0434669 | 5.49490263 |
| H | 6.59127547 | 3.88481786 | 5.61646861 |

**Supplementary Data 11.** Structure of the ***p*-2Urea** dimer (ii) with two AcO^–^ ions, S_0_ [DFT/CAM-B3LYP-GD3/3-21G, *E*_sp_ = -5032.13022304 hartree].

| atom | x[Å] | y[Å] | z[Å] |
| --- | --- | --- | --- |
| C | 11.3051515 | -5.0774174 | 0.4801143 |
| C | 10.5079601 | -5.8876535 | -0.409599 |
| C | 10.8081088 | -7.298666 | -0.4878078 |
| C | 12.1357336 | -5.7139435 | 1.39881963 |
| C | 12.2883567 | -7.1150532 | 1.42711624 |
| H | 12.7549349 | -5.1000229 | 2.04181326 |
| C | 11.6836044 | -7.8890664 | 0.4650215 |
| H | 12.9483605 | -7.5653352 | 2.16188442 |
| H | 11.8647091 | -8.9584107 | 0.41046284 |
| C | 9.43183655 | -5.3749364 | -1.2278382 |
| C | 10.2282156 | -8.0893532 | -1.5163345 |
| C | 9.34583927 | -7.5249089 | -2.4090319 |
| H | 10.4884863 | -9.1419353 | -1.5738727 |
| C | 8.9208725 | -6.1937961 | -2.2342335 |
| H | 8.09409582 | -5.8209612 | -2.8263523 |
| H | 8.9058943 | -8.1238725 | -3.2000581 |
| C | 11.4976313 | -3.6105654 | 0.31213014 |
| C | 11.7116444 | -2.7684269 | 1.41538316 |
| C | 11.7609344 | -3.0853834 | -0.9677517 |
| C | 12.2361676 | -1.488033 | 1.27168536 |
| H | 11.4692727 | -3.1306678 | 2.40952528 |
| C | 8.67827746 | -4.135781 | -0.9143585 |
| C | 12.5817806 | -1.0027258 | -0.0062504 |
| H | 12.4191328 | -0.8514457 | 2.12176635 |
| C | 12.2905892 | -1.8150793 | -1.1281221 |
| H | 11.58558 | -3.7092544 | -1.8359259 |
| C | 8.09328163 | -3.3568394 | -1.9299812 |
| H | 12.5366243 | -1.4320538 | -2.1118259 |
| C | 8.27309367 | -3.8785178 | 0.41222743 |
| C | 7.28701173 | -2.9500425 | 0.69646267 |
| H | 8.70874605 | -4.4610896 | 1.21557935 |
| C | 6.64603457 | -2.2223221 | -0.3397143 |
| H | 6.93855759 | -2.7946301 | 1.71117532 |
| C | 7.10645991 | -2.4174171 | -1.6626705 |
| H | 8.41370124 | -3.5041513 | -2.9571644 |
| H | 6.63188405 | -1.8499497 | -2.4467094 |
| N | 13.2275858 | 0.20960239 | -0.2529592 |
| N | 5.59636897 | -1.3960944 | 0.02372582 |
| C | 13.7184449 | 1.08686179 | 0.69574844 |
| H | 13.4178597 | 0.39825339 | -1.2801905 |
| C | 4.76154867 | -0.6807421 | -0.8388765 |
| H | 5.33364106 | -1.4503648 | 1.05375822 |
| O | 13.5466457 | 0.9730614 | 1.93560412 |
| O | 4.85353807 | -0.7094319 | -2.0909194 |
| N | 14.4515812 | 2.11233566 | 0.10290952 |
| N | 3.81743175 | 0.03413988 | -0.129444 |
| C | 15.091445 | 3.15203887 | 0.76141865 |
| H | 14.5966143 | 2.11829313 | -0.9519952 |
| C | 2.608851 | 0.53451372 | -0.6367581 |
| H | 3.9088891 | 0.10449859 | 0.92209288 |
| C | 15.0770434 | 3.36098716 | 2.15898679 |
| C | 2.23977239 | 0.51254526 | -1.9954359 |
| C | 15.8197131 | 4.07213552 | -0.0285911 |
| C | 15.7583177 | 4.43715186 | 2.71560019 |
| H | 14.5250287 | 2.65882547 | 2.76295504 |
| C | 0.96260162 | 0.91475569 | -2.3793818 |
| H | 2.96131621 | 0.14932552 | -2.7100186 |
| C | 1.66578652 | 1.01217502 | 0.29455542 |
| C | 16.4847692 | 5.35619592 | 1.93908657 |
| H | 15.7176184 | 4.56149056 | 3.79418319 |
| C | 16.4942151 | 5.14060673 | 0.55152154 |
| H | 15.8362364 | 3.91518374 | -1.1011686 |
| C | 0.39325126 | 1.40395297 | -0.1115991 |
| H | 1.9438473 | 1.02678519 | 1.34232283 |
| C | -0.0010601 | 1.35533219 | -1.4572549 |
| H | 0.70348386 | 0.8595612 | -3.4320044 |
| C | 17.2168004 | 6.52758174 | 2.61736336 |
| C | 17.9590994 | 7.42476915 | 1.59951312 |
| H | 17.2560976 | 7.85973308 | 0.88058559 |
| H | 18.4631714 | 8.24133187 | 2.13212603 |
| H | 18.7126729 | 6.84886554 | 1.05064857 |
| C | 18.2633598 | 5.97428776 | 3.6216151 |
| H | 18.9949052 | 5.35552408 | 3.09029265 |
| H | 18.7875453 | 6.79953819 | 4.1236776 |
| H | 17.7774405 | 5.35424569 | 4.38129021 |
| C | 16.1913779 | 7.41066928 | 3.37749273 |
| H | 15.4533108 | 7.80980987 | 2.67355372 |
| H | 15.6602274 | 6.82214823 | 4.13180433 |
| H | 16.7004068 | 8.24666105 | 3.87728967 |
| C | -1.4195847 | 1.72825706 | -1.9315604 |
| C | -1.3343523 | 2.92477732 | -2.9168316 |
| H | -0.7103509 | 2.67175629 | -3.7815958 |
| H | -2.3398701 | 3.18363323 | -3.2724731 |
| H | -0.8964104 | 3.79618016 | -2.4150928 |
| C | -2.0617294 | 0.5140133 | -2.6551495 |
| H | -1.4902077 | 0.25888893 | -3.5562143 |
| H | -2.058075 | -0.3525809 | -1.9926418 |
| H | -3.0973887 | 0.73005152 | -2.9413772 |
| C | -2.3406618 | 2.13792328 | -0.7589025 |
| H | -1.9657207 | 3.04378472 | -0.2657135 |
| H | -3.3470854 | 2.34075419 | -1.1406629 |
| H | -2.4241947 | 1.34296023 | -0.014639 |
| H | 17.0349109 | 5.81473294 | -0.1022388 |
| H | -0.3122738 | 1.71677455 | 0.64629888 |
| H | 3.14061154 | -1.5054501 | 4.78119836 |
| C | 4.05767503 | -0.9121722 | 4.67236651 |
| H | 4.87789636 | -1.4404419 | 5.16348639 |
| O | 5.05484272 | -1.698345 | 2.62602307 |
| C | 4.35226029 | -0.7682712 | 3.16686066 |
| H | 3.89106105 | 0.0722025 | 5.11524148 |
| O | 3.81328065 | 0.22060401 | 2.55851594 |
| H | 15.6147356 | 2.11928481 | -4.9649947 |
| H | 13.8528607 | 2.12828218 | -5.230914 |
| C | 14.6872421 | 1.57068792 | -4.7865476 |
| C | 14.4418655 | 1.43557816 | -3.2676016 |
| O | 13.6872784 | 0.47625177 | -2.8872228 |
| O | 14.9842553 | 2.31730902 | -2.509714 |
| H | 14.7160429 | 0.57803275 | -5.242522 |
| H | -13.797899 | 1.95738886 | 5.91428726 |
| H | -14.904525 | 0.56776554 | 6.23731439 |
| H | -13.136322 | 0.34826487 | 6.26203929 |
| C | -13.964066 | 0.88551177 | 5.78169535 |
| C | -13.969801 | 0.54514431 | 4.27531217 |
| O | -13.183184 | 1.22035962 | 3.52718311 |
| O | -14.735959 | -0.4122552 | 3.89758845 |
| H | 0.52644289 | -4.8425273 | 2.08140976 |
| H | 1.32099156 | -2.6358331 | 3.01434742 |
| H | -0.3701955 | -1.2976021 | 2.37358613 |
| H | -7.4689114 | 4.0690252 | 1.7400757 |
| H | -7.2328017 | 8.54178757 | 0.53888436 |
| H | -11.929807 | 2.593377 | 2.07174062 |
| H | 2.12476803 | -5.0325972 | 1.30523382 |
| H | 2.88302376 | -2.7823669 | 2.17080339 |
| C | -7.8910481 | 7.83433173 | 0.04491902 |
| H | -6.8425449 | 6.11983763 | 0.82151394 |
| C | 1.10559278 | -4.6476781 | 1.17054266 |
| C | 1.85111544 | -2.4284643 | 2.07500302 |
| C | -7.7007597 | 6.45461995 | 0.25193186 |
| H | -16.033035 | -2.1517383 | 3.15725636 |
| H | -10.737238 | 4.48904144 | 0.99848376 |
| H | -6.0815727 | 2.01510075 | 1.58847055 |
| H | -2.6443036 | -0.4526882 | 1.91053049 |
| C | -0.9008631 | -1.6741853 | 1.508752 |
| C | -7.370197 | 3.55472488 | 0.78881111 |
| H | -13.181899 | 0.79320928 | 1.95392271 |
| C | -11.805609 | 2.63277304 | 0.99566294 |
| H | -14.606383 | -0.721905 | 2.31655209 |
| O | -4.512817 | 0.53535938 | 1.36668233 |
| C | -8.8321131 | 8.27132098 | -0.8587996 |
| C | -2.1832096 | -1.1783792 | 1.25923819 |
| C | -6.6065875 | 2.39672087 | 0.7277091 |
| C | -11.14239 | 3.68871059 | 0.39100917 |
| H | 0.63365134 | -5.1820673 | 0.33794639 |
| C | 1.15233451 | -3.1228457 | 0.88635883 |
| H | 1.90345238 | -1.3469684 | 1.92988573 |
| H | -8.9250659 | 9.3242355 | -1.1071026 |
| H | -17.635754 | -4.0027721 | 2.93914624 |
| C | -16.227358 | -2.5985556 | 2.18879493 |
| C | -8.4990632 | 5.49140911 | -0.3636112 |
| N | -13.144385 | 0.64663067 | 0.90296425 |
| C | -0.2811395 | -2.6031565 | 0.66453364 |
| C | -8.0040785 | 4.09233627 | -0.346865 |
| N | -14.651421 | -1.0321132 | 1.29862211 |
| C | -17.132462 | -3.6446059 | 2.04885032 |
| C | -12.391777 | 1.60369781 | 0.22053523 |
| C | -9.6956678 | 7.3413921 | -1.4980319 |
| C | -9.6191577 | 5.93807172 | -1.1615669 |
| C | -4.7430781 | 0.05770853 | 0.22835295 |
| H | -19.612884 | -4.9934655 | 2.38059425 |
| C | -15.531798 | -2.0795162 | 1.07161085 |
| H | -18.300362 | -6.1665007 | 2.65155498 |
| H | 3.04913242 | -3.114244 | -0.205116 |
| C | -13.903068 | -0.3594045 | 0.33513843 |
| C | 1.99821664 | -2.8597264 | -0.3891855 |
| C | -11.031832 | 3.77470285 | -1.010074 |
| C | -2.8913536 | -1.5864841 | 0.1172457 |
| C | -6.4397663 | 1.7176594 | -0.5005421 |
| C | -19.05497 | -5.8245075 | 1.93470542 |
| C | -10.668597 | 5.0720416 | -1.644399 |
| C | -10.635104 | 7.78666614 | -2.4685686 |
| H | -10.648426 | 8.84001544 | -2.7322147 |
| N | -5.6353727 | 0.60764543 | -0.6924135 |
| N | -4.1176728 | -1.0528381 | -0.3022772 |
| C | -1.0228835 | -3.0375041 | -0.4499858 |
| C | -7.8876259 | 3.3740785 | -1.5550138 |
| C | -17.397755 | -4.2318515 | 0.80114616 |
| C | -12.206551 | 1.63838807 | -1.1767197 |
| H | -19.75235 | -6.6497777 | 1.74192878 |
| H | 1.63302465 | -3.4573011 | -1.2320769 |
| H | 1.93677941 | -1.8060689 | -0.6606205 |
| C | -2.2904212 | -2.5462008 | -0.7278043 |
| C | -7.1285214 | 2.21971913 | -1.6341035 |
| C | -11.551581 | 5.56256201 | -2.6029446 |
| C | -11.544134 | 2.70917444 | -1.7679217 |
| C | -11.507334 | 6.89816973 | -3.0513557 |
| C | -15.792715 | -2.6646171 | -0.1881015 |
| O | -13.934972 | -0.6290505 | -0.8921651 |
| C | -18.39196 | -5.3902592 | 0.60666954 |
| C | -16.701661 | -3.7099773 | -0.3026934 |
| H | -0.5825172 | -3.7511703 | -1.1384286 |
| H | -8.3617019 | 3.76727254 | -2.4466291 |
| H | -20.049573 | -4.0883612 | 0.04437626 |
| H | -12.355527 | 4.91760335 | -2.936732 |
| H | -5.5378144 | 0.30262283 | -1.7092216 |
| H | -4.4792321 | -1.4551992 | -1.2100076 |
| H | -12.617425 | 0.8324768 | -1.7623701 |
| H | -12.218025 | 7.23392362 | -3.7999589 |
| H | -16.857799 | -6.9375766 | 0.70325973 |
| C | -19.516083 | -4.9510508 | -0.3696941 |
| H | -15.26526 | -2.268549 | -1.0410054 |
| C | -17.649376 | -6.6189713 | 0.01660972 |
| H | -2.8265326 | -2.8403188 | -1.623507 |
| H | -6.9906951 | 1.70159786 | -2.5765282 |
| H | -11.427712 | 2.72660347 | -2.8470255 |
| H | -20.229078 | -5.7721532 | -0.5289907 |
| H | -18.348544 | -7.4527215 | -0.1378304 |
| H | -16.869572 | -4.1315236 | -1.2899361 |
| H | -19.093818 | -4.6608903 | -1.3367397 |
| O | -4.7413573 | -2.0885953 | -2.7095831 |
| O | -5.3584669 | 0.03875859 | -3.2829572 |
| H | -17.187757 | -6.3662727 | -0.9428337 |
| C | -4.984707 | -1.1623934 | -3.5569115 |
| C | -4.7376237 | -1.4767604 | -5.0456001 |
| H | -5.4174646 | -0.88883 | -5.6665939 |
| H | -4.853094 | -2.5477528 | -5.2261352 |
| H | -3.7064749 | -1.1868951 | -5.2856664 |
